# Exploring young women's experiences of a mindful yoga intervention for depression in the Netherlands: Qualitative analysis of positive and negative effects

**DOI:** 10.1111/bjc.70013

**Published:** 2025-09-17

**Authors:** Nina K. Vollbehr, Brian D. Ostafin, Agna A. Bartels‐Velthuis, H. J. Rogier Hoenders

**Affiliations:** ^1^ Center for Integrative Psychiatry Lentis Psychiatric Institute Groningen The Netherlands; ^2^ Department of Clinical Psychology and Experimental Psychopathology University of Groningen Groningen The Netherlands; ^3^ University Center for Psychiatry, Rob Giel Research Center University of Groningen, University Medical Center Groningen Groningen The Netherlands; ^4^ Department of Religion, Culture and Society University of Groningen Groningen The Netherlands

**Keywords:** adverse effects, depression, qualitative measures, yoga, young women

## Abstract

**Objectives:**

Evidence of the benefits of yoga for patients with major depressive disorder (MDD) is mixed and results mainly from randomized controlled trials (RCTs). Adding qualitative measures to RCTs may give additional insight into the range of outcomes experienced by participants. We therefore used qualitative measures to assess the positive and negative experiences of young women (18–34 years) with depression who received a 9‐week mindful yoga intervention added to treatment as usual.

**Methods:**

We conducted qualitative interviews after a 12‐month follow‐up alongside an RCT in the Netherlands. Questions were open‐ended and assessed experiences with mindful yoga reflecting positive or negative experiences. In addition, we explicitly asked about negative effects. Interviews were systematically analysed, and statements were placed in five domains (Affective, Cognitive, Conative, Somatic and Yoga Skills) and diverse subcategories.

**Results:**

We collected qualitative interviews of 58 of the 88 participants randomized to mindful yoga (66%). The majority of participants had no previous experience with yoga (76.8%). Mean age was 24.68 (*SD* = 4.70). A little over half of the participants were employed (53.6%). All participants were diagnosed with current depression. Level of self‐reported symptoms of depression was considered ‘severe’ and level of clinician‐rated symptoms of depression was considered ‘moderate’. For most participants, the current episode started 1–2 years ago (35.8%) or more than 2 years ago (34%). Of these 58 participants, 81.0% reported positive effects and 65.5% reported negative effects. Positive experiences consisted mostly of positive affect (56.9%), meta‐cognition (44.8%) and general physical relaxation (41.4%). Negative experiences consisted mostly of meta‐cognition (37.9%), agitation or irritability (20.7%) and physical inflexibility (12.0%).

**Conclusions:**

Most young women in the mindful yoga intervention experienced both positive and negative effects. In future research, broader measurements of positive effects and structural assessments of negative effects are warranted.

**Practitioner points**

- Individuals with MDD might experience a range of positive and negative effects during a mindful yoga intervention.
- Positive effects include positive affect, meta‐cognition and general physical relaxation; negative effects include meta‐cognition, agitation or irritability and physical inflexibility.
- Experiencing more negative, challenging effects might lead to more intervention drop‐out.
- Offering counselling during the initial phase of the intervention might offer participants skills to cope with difficult experiences and reduce the number of drop‐out.

## INTRODUCTION

The advantages of yoga for individuals with major depressive disorder (MDD) are mixed, as a meta‐analysis of 18 randomized controlled trials (RCTs) shows (Vollbehr et al., 2018). The results of our RCT on the efficacy of adding a mindful yoga intervention to treatment as usual for young women with MDD did not find additional benefits regarding the reduction of symptoms of depression or diagnostic status (Vollbehr et al., 2018), which is in line with the meta‐analysis findings. The RCT is still considered the gold standard of research; however, RCTs are criticized for limitations such as their focus on analysing group means, with the consequence that differences between individuals in treatment effects can get obscured (Hamaker et al., 2005). In addition, with its focus on statistical differences in a certain population, the individual patients, with their individual circumstances, needs and wishes can get lost in the analyses, making it difficult to tell whether a treatment that was found effective in a specific population is also effective for specific individuals (Deaton & Cartwright, 2018). To address this issue, researchers have started to encourage adding qualitative measures to RCTs (Davis et al., 2019).

Qualitative measures can give additional insight into the range of outcomes experienced by participants (O'Cathain et al., 2014), which is especially important when it comes to yoga interventions and potential negative effects. In yoga research, only just over half of the studies, 54%, include drop‐out rates or adequately report safety data including adverse events (Cramer et al., 2015, 2016). Reporting on these data would provide important information to adequately interpret the effects of yoga interventions and would be especially important to understanding the experiences that lead to drop‐out of participants. To draw a parallel with meditation research, potential adverse effects of meditation practice have only recently gained more attention. A survey of Western practitioners of Buddhist meditation suggests some preliminary evidence of a diverse range of meditation‐related challenges (Lindahl et al., 2017). The initial findings of Lindahl et al. (2017) indicated that adverse experiences are rather common—that is, all practitioners reported meditation‐related challenges such as sleep changes, depression, dysphoria or grief, or social impairment. These data should be interpreted with caution, as only 60 practitioners were included. However, these results suggest that meditation, and therefore perhaps yoga practices too, might come with (mental) health challenges.

Building on these findings for meditation challenges, studies of an 8‐week mindfulness intervention have found that practitioners report a relatively high number of momentary unpleasant experiences (e.g., 66%, Baer et al., 2021; 87%, Aizik‐Reebs et al., 2021). Most unpleasant experiences are transient (25% report unpleasant experiences lasting after the training, Aizik‐Reebs et al., 2021); with a minority experiencing harm (defined as being ‘worse off, in any way, after the course, than you would have been if you hadn't done the course’) from the training (4%–7%, Baer et al., 2021). These two studies used samples from the general population. Given that mindfulness training is also taught for a range of mental conditions, these rates might be higher in clinical samples.

In the field of mindfulness/meditation research, there is debate regarding what constitutes an adverse practice‐related effect. Some argue that adverse events during mindfulness training are events which lead to severe outcomes or hospitalization and that mild adverse events such as anxiety and pain are expected, transient and might be part of the process of gaining benefit from the practice (Binda et al., 2022). The benefit from mindfulness training would be developing more mindfulness. As a definition of mindfulness, two components are highlighted (Bishop et al., 2004). The first component encompasses increased attentional regulation, maintained on the present moment, with enhanced awareness of direct experience (e.g., monitoring skills). The second component includes an attitude of curiosity, openness and acceptance towards these experiences (e.g., acceptance skills). Both aspects and skills are important in gaining benefits from mindfulness interventions. However, it is hypothesized in the monitor and acceptance theory that enhanced awareness of experience without an accepting attitude might increase affective reactivity, intensifying negative affective states (Lindsay & Creswell, 2017). Research has found that monitoring skills generally develop before acceptance skills (Baer et al., 2012), making it likely that practitioners will experience increased awareness of discomfort in the early stages of training. Evidence supports this idea, as studies assessing negative experiences of participants in a mindfulness intervention found that difficult emotions, cognitions, sensations, thoughts, memories, pain or discomfort were frequently reported as a negative experiences resulting from practicing mindfulness (Baer et al., 2021).

As yoga interventions often involve mindfulness training (Anderson & Sovik, 2000), we would expect yoga practitioners to experience a range of unpleasant events. Several studies have investigated the safety of yoga and the experience of adverse events during yoga interventions (Cramer et al., 2015, 2017, 2019). However, studies use many different definitions of adverse events (Cramer et al., 2017), making it difficult to adequately assess potential adverse events in yoga practice. In addition, most research of adverse events is conducted in the general population (Cramer et al., 2017, 2019), making it impossible to draw conclusions regarding yoga interventions for individuals with mental health conditions.

To date, for individuals with MDD, only two studies used qualitative assessments alongside an RCT to explore experiences with the yoga intervention (Kinser, Bourguignon, Taylor, & Steeves, 2013; Uebelacker, Kraines, et al., 2017). We found another two studies exploring experiences of individuals with MDD in an open trial (Capon et al., 2021; Lee et al., 2019). Although one of these four studies assessed ideas to improve the programme (Uebelacker, Tremont, et al., 2017) and another assessed boundaries towards practicing yoga (Lee et al., 2019), none of these studies assessed adverse events or negative experiences of yoga participants. Regarding positive effects, both RCTs did not find a significant difference between the yoga group and the control group in reduction of symptoms of depression immediately after the intervention (Kinser, Bourguignon, Whaley, et al., 2013; Uebelacker, Tremont, et al., 2017), but the qualitative assessment found some indication of beneficial experiences for participants. Kinser, Bourguignon, Taylor, and Steeves (2013) found that participants reported (1) developing a skill for managing symptoms of depression and stress, (2) getting out of their heads and experiencing some control, (3) finding courage to undertake more activities, (4) gaining a new narrative about depression (i.e., separating themselves from their depression), (5) being honest with themselves and others about their feelings, (6) feeling okay with who they are. In the study of Uebelacker, Kraines, et al. (2017) participants were asked about the most important thing that they learned, which were (1) to focus, concentrate and be in the present moment, (2) relaxation skills, (3) self‐acceptance, (4) more self‐awareness or body awareness, (5) better coping skills with negative thoughts, (6) how to quiet their minds, (7) to centre or ground themselves and (8) to decrease negative reactivity. These positive experiences paint a broader picture of potential effects of a yoga intervention than quantitative assessments of decreased symptoms of depression.

However, these studies are focused on adults with depression (mean ages resp. 43.26 and 46.78 years) and our RCT was focused on young adults (mean age 25.0 years). In this light, it is important to note that in the Aizik‐Reebs et al. (2021) study, the mean age was 25.05. Given that 87% of young adults from the general population in the Azik‐Reebs et al. study experienced negative effects from the mindfulness training, this could even be higher for young adults with MDD. In addition, it might be the case that young adults with depression even need further specialized care in addition to mindful yoga, compared to young adults from the general population. This is another reason for further examining experiences in this group.

### Current study

Given the lack of information regarding yoga interventions and potential negative effects, as well as a broader overview of the range of experiences of participants, we have conducted a qualitative analysis in addition to our RCT. The main purpose was to gain more in‐depth insight into the experiences of young women with MDD following a mindful yoga training added to treatment as usual. We therefore added qualitative interviews to our RCT assessing both positive and negative experiences. We defined a negative experience as an experience that was ‘unexpected, challenging, or difficult’, following Lindahl et al. (2017). We followed the reports of the women, how they described their experiences and whether they attributed this experience to practicing yoga, without interfering with it (e.g., labelling it as needed to gain benefits from the practice or as expected from practicing mindful yoga).

## METHODS

### Study design

The Medical Ethical Committee of the University Medical Center Groningen granted ethical approval of the study protocol (registration number NL.59324.042.16/2016/533). A more detailed description of the design, procedure and assessments is published elsewhere (Vollbehr et al., 2020). This qualitative analysis is reported following the Standards for Reporting Qualitative Research (O'Brien et al., 2014) and the Journal Article Reporting Standards for Qualitative Research (Levitt et al., 2018). We used a phenomenological approach focusing on the subjective experiences of the participants of the intervention (Harper, 2011).

N.K.V., a PhD student, *Yoga Alliance Registered Yoga Teacher* ^*®*^ *500* and psychologist, oversaw the supervision of the research assistants conducting the interviews. B.D.O. is a professor of clinical psychology and experimental psychopathology with expertise in mindfulness research. A.A.B.V., a senior researcher in psychiatry, was one of the two interview raters and was involved in the adaptations of the code book. H.J.R.H. is a professor of lifestyle medicine in psychiatry with an expertise in lifestyle and mind–body interventions research.

### Participants

For the RCT, 171 young women (age 18–34) with a primary diagnosis of MDD, currently receiving treatment at various psychiatry outpatient clinics in the northern part of the Netherlands, were recruited. Eighty‐eight participants were randomized to receive the mindful yoga intervention added to their treatment as usual; the other 83 participants received treatment as usual only. The first author (N.K.V.) was involved as the teacher of the mindful yoga intervention and met the participants during the sessions. The other authors (B.D.O., A.A.B.V., H.J.R.H.) did not have any contact with the participants.

### Procedure

Participants were recruited from January 2017 to October 2018. After informed consent, the research assistant administered a structured interview for DSM‐IV (SCID‐I; First et al., 2002) to verify MDD diagnosis and inclusion and exclusion criteria, before inviting the participant to enter the trial. Assessments were at baseline (before randomization), post‐intervention (10–12 weeks post‐baseline), and 6 and 12 months after post‐intervention. Demographics were assessed during the first assessment. Qualitative interviews were administered by phone after completion of the last assessment to not intervene with the randomization and quantitative measures. The research assistant conducting the assessments was blind to condition assignment. During the last assessment of the RCT, she scheduled an appointment for the qualitative interview with the participant. Before this final interview the randomization was broken, as only mindful yoga participants were interviewed about their experiences with the intervention (but all participants were interviewed about their experiences in the study). Randomization was broken by the project coordinator (N.K.V.) giving a list of condition assignments to the research assistant. After this, the research assistant interviewed the participant.

### Ethical considerations

The research assistant conducting the assessment was also a young adult. She was involved in all assessments with all participants, starting from the screening and baseline to post‐intervention and the follow‐up assessments. The screening assessment was over the phone, the baseline and post‐intervention assessments in person and the follow‐up assessments over the phone. Interviews lasted between 10 and 30 min. During the study, the research assistant built relationships with the participants. Participants talked about symptoms, their lives and experiences, and the research assistant was an active listener (and coder of the symptoms). In this light, it is important to consider several ethical issues that come up with this research method.

First, participants all provided informed consent for the study. Before providing consent, they were explained everything about the study, including potential risks and benefits. Potential additional questions were answered, and they were given time to think it over, talk it through with a closed one, or consult an independent physician. Second, all data were handled confidentially in compliance with the Dutch Personal Data Protection Act (in Dutch: De Wet Bescherming Persoonsgegevens; WBP). Each participant received a unique ID code, and all assessment data were coded using this number. Third, confidentiality was discussed with the participant, including that none of their data or information were shared with others (except when coded in their anonymous form). Their therapists were only told whether the participants were receiving mindful yoga or not. Fourth, the researcher–participant relationship inherently contains a power imbalance. We therefore cannot rule out that this might have influenced participants' responses. Fifth and finally, given the multiple assessments and emotionally sensitive topics that participants were asked to disclose (i.e., depression symptoms), there might have developed some therapeutic dimension in the relationships between participants and the research assistant. In case important issues came up (including suicidality) the research assistant always referred participants to their own therapist. When necessary, the research assistant discussed with the participants about informing their therapist in order to ensure maximum access to care.

### Mindful yoga intervention

The mindful yoga intervention was a manualized 9‐week group training with 1.5‐h weekly sessions. Detailed information about the intervention has been published elsewhere (Vollbehr et al., 2021). The intervention contained yoga postures, breathing and meditation practices (Anderson & Sovik, 2000), in combination with instructions intended to increase mindful awareness. For instance, the instructor gave participants cues to focus on present moment experience (e.g., their breath or bodily sensations) and encourage self‐compassion (e.g., inviting them to listen to the limits of their body and telling them to come out of the posture when needed). The intervention was designed to enhance awareness of body sensations, emotions and thoughts, and change processes that contribute to MDD (Vollbehr et al., 2020).

To create safety within the group setting, additional instructions consisted of (1) inviting participants to open their eyes whenever necessary, (2) refraining from individual feedback or hands‐on adjustments, (3) encouraging the use of yoga props (e.g., cushions, blankets, blocks), and (4) giving adaptations of yoga postures when appropriate (e.g., doing a standing posture seated for participants having difficulty standing). In order to encourage home practice, participants conducted an online module that consisted of psychoeducation about depression, self‐monitoring assignments and videos for home practice (recommended time: 30–45 min a day). The information and videos could be accessed at all times during the intervention. However, in order to go from the first session to the next, the participant had to complete the first session. After completion, a new session became accessible, without losing access to the first session.

### Treatment as usual

TAU was delivered as an individualized standard of care (Freedland et al., 2011) and administered according to the Dutch Treatment Guideline (Spijker et al., 2012). A more detailed description of the treatment in the study has been published elsewhere (Vollbehr et al., 2022).

### Qualitative interview

Table 1 shows the questions of the qualitative interview. The first author (N.K.V.) designed the questions under the supervision of the other authors (B.D.O., A.A.B.V., H.J.R.H.). Question five was an open‐ended question about potential effects, that could be answered with effects that were experienced as positive or negative. With question six we explicitly asked about experiences labelled as ‘unexpected, challenging, or difficult’. The next sub‐questions were asked when a participants answered this sixth question affirmative. We took these questions from the Lindahl et al. (2017) interview.

**TABLE 1:** Questions of the qualitative interviews.

	Question
1.	Can you tell us about your experiences with yoga during and after the mindful yoga intervention? *Can you tell us more about that? Can you explain that?*
2.	What is different for you from the time you started mindful yoga to now? *How has this influenced your depressive symptoms? Can you tell us more about that? Can you give an example?*
3a.	What did you learn in the mindful yoga training? *Can you tell us more about that? Can you give an example?*
3b.	Are there things you have learned that you apply in your daily life? *Can you tell us more about that? Can you give an example?*
4.	What role does yoga have in coping with your depression or in your recovery? *Can you tell us more about that? Can you explain that? Can you give an example?*
5.	Did you notice any effects during and after training? If so, what kind of effects? *What have you noticed about your body? What have you noticed in other areas of your life? How did the training affect your depressive symptoms? How did the training affect how you dealt with your depressive symptoms?* *Can you tell us more about that? Can you explain that? Can you give an example?*
6.	Have you had any striking experiences through yoga that were unexpected, challenging, or difficult? *If yes: how much did you practice at that time? What were your living conditions at that time? What impact have these experience(s) had on your life? What is the reason you associate this experience(s) with yoga? How have you interpreted this experience(s)? How have others around you interpreted this experience(s)? How have you reacted to this experience(s)? What did and didn't help you to deal with this experience(s)?*

### Data processing

Interviews were digitally recorded and audio files were transcribed by Clinical Psychology master students. Interviews were transcribed verbatim, with vocalizations such as ‘um’, ‘you know’ and ‘so’ removed. The first author (N.K.V.) verified transcripts to ensure an accurate description of content.

### Data analysis

Following Lindahl et al. (2017), we used qualitative data analysis to systematically organize and describe data retrieved from interviews. We used deductive thematic analysis (Elo & Kyngäs, 2008) with the phenomenological codebook by Lindahl et al. (2017) as a theoretical framework to guide our analysis. This codebook contains seven domains that each have subcategories: Affective (13 subcategories), Cognitive (10 subcategories), Conative (3 subcategories), Perceptual (7 subcategories), Sense of self (6 subcategories), Social (5 subcategories) and Somatic (15 subcategories). A categorization matrix with these domains and subdomains as categories was made and the data were coded for correspondence with these categories. Two independent raters (A.A.B.V. and a nurse practitioner in training) coded the interviews. They discussed their scoring until they reached agreement. If they did not reach agreement or needed additional discussion to find agreement, they consulted a third rater (N.K.V.) until agreement was reached. After all interviews were coded, N.K.V. also verified all scorings to ensure accuracy.

During the coding, we noticed that only some of the categories of Lindahl's codebook were useful for our sample and many experiences were coded as ‘other’, not corresponding with the original description of the categories. We noticed that most experiences reported by our participants were within the ‘normal’ range of experience than was meant in Lindahl's codebook. For example, many participants reported a general feeling of relaxation, but not a release of tension in specific areas of the body (i.e., knots) as was described in the codebook (category *release of tension*). In the somatic domain, we found that several categories only focused on more problematic experiences (such as more headaches, more cardiac irregularities) where our participants also reported experiences of decreased problematic somatic experiences (i.e., fewer headaches, the absence of cardiac irregularities). Finally, we found several recurring statements that were not captured by Lindahl's codebook. Therefore, we decided to use a data‐driven approach regarding the interview content coded as ‘other’, following the original study by Lindahl et al. (2017), using interview content to adapt the codebook categories. Using the Lindahl et al. codebook as a draft, after the first ten interviews, the two independent raters and the third rater discussed the statements coded as ‘other’ and adapted the categories of the original codebook to encompass these statements. After this, coded interviews were recoded in the new structure. After the next ten interviews, this process was repeated. After coding and discussing thirty interviews, no additional changes were made and the codebook was finalized, capturing the changes from the Lindahl et al. codebook based on our data.

Our adapted version of the codebook consists of five domains with subcategories: Affective (experiences regarding changes in emotions; six subcategories), Cognitive (experiences related to changes in mental functioning and cognitive processes; three subcategories), Conative (experiences regarding changes in motivation or goal‐directed behaviours; three subcategories), Somatic (experiences related to bodily functioning or physiological processes; eight subcategories) and Yoga Skills (a domain that we added to the original domains because it included experiences that were not covered in the other domains; four subcategories). A complete description of our codebook can be found in Appendix S1.

## RESULTS

**FIGURE 1:**
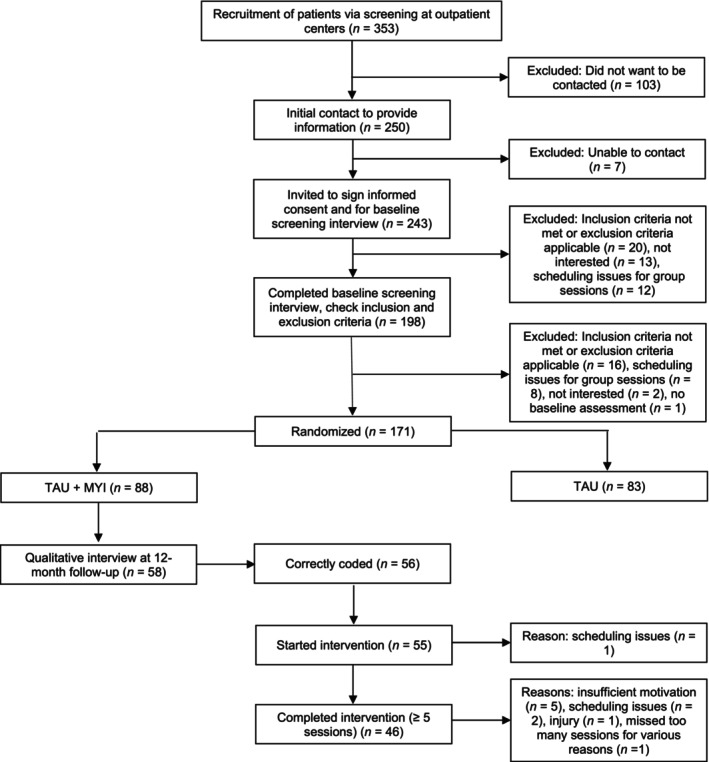
Participant recruitment and flow through the study.

An overview of participant flow can be found in Figure 1. We were able to collect interviews of 58 (66%) of 88 participants randomized to mindful yoga. There were no significant differences between participants that did and did not complete the interview regarding (a) symptoms of depression at baseline (*p*s ≥ .243) or 12‐month follow‐up (*p*s ≥ .597), (b) diagnosis of depression at 12‐month follow‐up (*p* = .795), (c) experience with yoga at baseline (*p* = .263), (d) motivation to study participation at baseline or 12‐month follow‐up (*p*s ≥ .132), (e) reported positive effects at post‐intervention (*p* = .064), (f) evaluation of the quality of the mindful yoga training and teacher (*p*s ≥ .409), or any of the (g) clinical (*p*s ≥ .287) or (h) demographic measures (*p*s ≥ .137). Nor did interview completers finish the yoga intervention more often (*p* = .387). The only significant difference was the amount of yoga practice at the post‐intervention (*p* = .033) with interview completers reporting a higher level of practice than non‐completers. This difference was not present at the follow‐up assessments (all *p*s ≥ .594). Demographic and clinical information about the 58 participants can be found in Table 2.

**TABLE 2:** Participants' demographic and clinical characteristics at baseline.

Variable	Completed interview (*n* = 56)
Age, years, *M* (*SD*)	24.68 (4.70)
Symptoms of depression at baseline, *M* (*SD*)	
Clinician rated (HDRS)	19.36 (5.86)
Self‐reported (DASS‐sf)	22.32 (9.31)
Number of previous episodes^a^, *n* (%)	
0	17 (31.5)
1–2 episodes	27 (50.0)
3 or more	10 (18.5)
Duration current episode^b^, *n* (%)	
2 weeks–3 months	1 (1.9)
3–6 months	5 (9.4)
6 months–1 year	10 (18.9)
1–2 years	19 (35.8)
2 years or more	18 (34.0)
Motivation for study, *M* (*SD*)^c^	7.80 (1.00)
Experience with yoga, *n* (%)	
Yes	13 (23.2)
No	43 (76.8)
Relationship, *n* (%)	
Yes	28 (50.0)
No	28 (50.0)
Children, *n* (%)	
Yes	7 (12.5)
No	49 (87.5)
Paid job, *n* (%)	
Yes	30 (53.6)
No	26 (46.4)
Currently in school, *n* (%)	
Yes	30 (53.6)
No	26 (46.4)
Completed the intervention^a^, *n* (%)	
Yes	46 (82.1)
No	10 (17.9)

Abbreviations: DASS‐sf, Depression Anxiety Stress Scales, short form; HDRS, Hamilton Depression Rating Scale.

^a^ *n* = 54.

^b^ *n* = 53.

^c^ Scale 1–9.

Of the 58 participants who completed the interview, for two participants the subject number was wrongly coded when saving the audio file of the interview, resulting in missing additional information regarding demographics and number of sessions completed. The majority of participants had no previous experience with yoga (*n* = 43, 76.8%). Of the remaining 56 participants, one participant did not start the intervention due to scheduling issues. Of the 55 remaining participants, 46 completed five or more sessions of the 9‐week mindful yoga intervention (79.3%). Reasons for non‐completion were insufficient motivation (*n* = 5), scheduling issues (*n* = 2), injury unrelated to yoga (*n* = 1) and various reasons for missing a session (*n* = 1). Mean number of completed sessions of the remaining 46 participants was 6.91 (*SD* = 1.23, range 5–9). Mean age of the participants was 24.68 (*SD* = 4.70). Half of the participants were in a relationship (*n* = 28), and the other half were not (*n* = 28). A minority had children (*n* = 7, 12.5%). A little over half of the participants were employed (*n* = 30, 53.6%). All participants were diagnosed with current major depressive disorder using the SCID‐I interview (First et al., 2002). Level of self‐reported symptoms of depression was considered ‘severe’ (Lovibond & Lovibond, 1995) and level of clinician‐rated symptoms of depression was considered ‘moderate’ (Zimmerman et al., 2013). The majority of participants reported one or two previous episodes of depression (*n* = 27, 50%). A minority reported it was their first episode (*n* = 17, 31.5%) or reported three or more previous episodes (*n* = 10, 18.5%). For most participants, the current episode started 1–2 years ago (*n* = 19, 35.8%) or more than 2 years ago (*n* = 18, 34%).

Of the 58 participants, 81.0% reported *any* positive effects, and 65.5% *any* negative effects. Half of the participants reported *both* positive and negative effects, 18 participants (31.0%) reported *only* positive effects, nine participants (15.5%) reported *only* negative effects, and two participants (3.4%) reported no effects. Participants reporting only positive effects or both positive and negative effects were, compared to participants reporting only negative effects or no effects, more likely to have completed the training (*p* < .001), report a higher motivation to participate in the study at post‐intervention and 6‐month follow‐up (*p*s ≤ .001/.046; not the other assessments, *p*s > .164), report positive effects of the yoga training at all assessments (*p*s ≤ .01), give higher ratings to the yoga training and teacher (*p*s ≤ .035) and practice yoga at post‐intervention and 6‐month follow‐up (*p*s = .001/.017; not at 12‐month follow‐up, *p* = .096). There were no differences on any of the clinical (*p*s ≥ .341) or demographic measures (*p*s ≥ .168).

### Positive effects

For a complete list of statements and corresponding domains and subcategories, see Appendix S2. One statement is given as an example for the categories that were most reported.

#### Affective domain

Positive affect was most reported (*n* = 33, 56.9%). Statements referred to states of positive affect with a low level of arousal, such as calm and peace: ‘I have found more peace [because of the intervention]’ or positive affect with more (pleasant) arousal, such as energized or feeling productive: ‘Each time I went [to the yoga class I felt] calmer and recharged […], more energized’. One participant reported crying or laughing.

#### Cognitive domain

Meta‐cognition was the second most‐reported category (*n* = 26, 44.83%). Statements included becoming more aware of themselves: ‘[I learned] self‐reflection […]. You are constantly confronted with yourself [in the yoga intervention]. That self‐reflection that you can do certain things differently’. Of thoughts: ‘I notice that I can separate my thoughts more from myself, so that I am not my thoughts […]’. And of feelings: ‘I have become more aware of my body and the things I feel. I have become more aware of my feelings. When someone asks me how I'm doing, I used to tend to say “I'm fine”, but now I can think about it better and answer more honestly’.

Twelve participants (20.7%) reported mental stillness. Statements referred to experiencing a calmer mind or being not so focused on what is going on in their minds: ‘It's […] the peace it [the yoga intervention] gave me. I am very, very busy in my mind and at that moment [of the yoga class] I could really calm down in my mind […]’. Change in executive functioning, especially concentration, was reported by two participants.

#### Conative domain

Twenty participants (34.5%) reported change in effort or striving in daily life. Statements referred to taking more time for rest: ‘If I now notice that I've done too much again, I'll take my moment of rest’. More time for focusing on themselves and how they feel: ‘[I've learned] to take a break and notice “how do I feel now? Do I still feel like doing this? Is it still okay?”. More often [I take] a moment to evaluate instead of just continuing [what I am doing]’. More time for self‐care: ‘I've given myself more time to slow down. [Before the yoga intervention I] just kept going and going and going’. Also, pushing themselves less and listening to their limits was reported: ‘I am very hard on myself. And I learned […] in yoga that less is also allowed’. Change in motivation or goal in daily life was reported by three participants.

#### Somatic domain

General physical relaxation was reported by 24 participants (41.4%). Most statements referred to a general state of relaxation: ‘Doing yoga […] made me feel […] relaxed’. Some statements referred to a specific relaxation of the body: ‘[The yoga intervention brought me] relaxation. Getting out of your mind for a while. Focusing on your body. [After that] you don't have everything so tight anymore’. Eight participants (13.8%) reported breathing changes: ‘I never really thought about the fact that I was breathing faster. Now I notice that if I pay attention to that and consciously go there [my breathing] for a while, it goes a bit better’. Cardiac changes, (reduction of) headaches or head pressure, pain reduction and sleep changes were reported by one participant.

#### Yoga skills domain

In this domain, body awareness was most reported (*n* = 21, 36.2%). Statements referred to a general awareness of or listening to the body: ‘[I've learned that I] have to better listen to my body. That [my body] says a lot. Listen to your body and then notice that something is wrong’. Some participants described strategies they learned to better cope with bodily signals: ‘[After the yoga intervention], I felt that it helped me to feel pain in my body I wasn't aware of before. To notice more [inner] signals. To be able to breathe towards the pain […]’. Few participants gave examples on how they learned to listen to their bodies, for instance by respecting physical limitations: ‘[I learned in the yoga intervention] that you should not go beyond what your body indicates. With stretching exercises don't go too far. If you do, you have to go back a bit. I apply that now. I don't go that far beyond my limits anymore. Physical and mental limits. I won't go that far beyond those anymore’.

Acceptance was reported by 20 participants (34.5%). Statements referred to self‐acceptance: ‘I got frustrated very quickly and [was] very punishing [on myself]. I'm working on that now [since the yoga intervention]. I'm thinking “it's good that I'm here and I'm doing what I can; it doesn't have to be perfect”’. Acceptance of thoughts or feelings: ‘[I've learned] that bad thoughts […] can just be there. More acceptance. That it's okay to feel certain things. That helps. That you acknowledge that it [bad thoughts] is there’. And acceptance of other things such as situations or events: ‘[I've learned] that it's fine if you just notice something. You don't have to change it right away. Not all the negative things are something you have to do something about. You can just notice it and let it be’.

Seven participants (12.0%) reported self‐compassion: ‘[Because of the yoga intervention] I try to be less hard on myself. Before, if I made a mistake or if something went wrong, I could get very angry. And now I think “okay, it's done, how do we proceed”’. Six participants (10.3%) reported more mindfulness: ‘I try to be a bit more in the here and now in daily life […]’.

#### Other

Six participants reported positive effects that did not fit within a domain and were scored as ‘other’. Two statements referred to positive experiences from being in a group with other women with depression. The other four statements referred to having learned to better deal with frustrations, using a posture to feel more present, having more patience with a child and behavioural activation.

### Negative effects

See Appendix S3 for a complete list of statements and the corresponding domains and categories. One statement is given as an example for the categories that were most reported.

#### Affective domain

Twelve participants (20.7%) reported agitation and irritability, for example being restless: ‘I couldn't handle the peace in the training. I noticed that I became more restless from the quiet [atmosphere] and had trouble staying seated’. Getting frustrated was also reported: ‘During the training I often felt frustrations about things not working out for me. I found that confrontational and less enjoyable’. As were statements of feeling impatient and waiting for it to be over: ‘It [the yoga intervention] was mostly lying still [which I didn't like] and watching your breathing for 10 min and things like that. In my mind, I had [thoughts] like “aren't we finished yet?”’.

Self‐conscious emotions were reported by six participants (10.3%). Statements concerned being in the group with other women who were depressed: ‘[The yoga class] was a social thing because everyone started doing it at the same time. But [there was] no real talking, so [it] actually felt really awkward’. Also, feeling ashamed or uncomfortable was reported: ‘[We did an exercise in which] you bend over and have to let go and sigh very loud. I just couldn't do that. Because I felt ashamed. I struggled with that’. Rage, anger or aggression and re‐experiencing of traumatic memories were reported by one participant.

#### Cognitive domain

Meta‐cognition was most‐reported (*n* = 22; 37.9%). Statements were about noticing difficult things, such as difficulty concentrating or mind wandering: ‘[During the yoga classes] you had to concentrate. [When that happens] my mind starts thinking about other things a lot and I wasn't relaxed at all’. Noticing difficult thoughts and feelings were also mentioned: ‘[During the yoga classes] I suddenly really noticed all the pain [in my body]. […] I felt fatigue in my body. [I was] really exhausted’. Becoming aware of tendencies that are not helpful was also reported: ‘[During the yoga intervention I noticed] that I thought quite negatively about myself’.

#### Conative domain

One participant reported a change in effort or striving.

#### Somatic domain

Seven participants (12.1%) reported inflexibility. Statements concerned having physical difficulties performing the postures: ‘Physically, [it was] very difficult […] at times. I couldn't participate in some things’. Six participants reported pain (10.3%), with three statements referring to sore muscles after a yoga classes. The other statements were about other forms of pain. Two participants reported fatigue or weakness and one participant reported breathing changes.

#### Other

One participant reported being uncomfortable when having eyes closed. This statement did not fit into a category; therefore, we labelled this as ‘other’. However, it is important to note that participants were not instructed to keep their eyes closed, this was offered as a possibility, they were always encouraged to keep their eyes open whenever eyes closed was uncomfortable.

## DISCUSSION

In our qualitative assessment, we found that most participants of a 9‐week mindful yoga intervention reported both positive and negative effects. Positive effects that were reported most were positive affect, meta‐cognition, general psychological relaxation, body awareness, acceptance, change in effort or striving in daily life, and, to a lesser extent, mental stillness, breathing changes, self‐compassion and mindfulness. Most‐reported negative effects were meta‐cognition, agitation or irritability, inflexibility, pain and self‐conscious emotions. These results provide new knowledge regarding young adult women with MDD following a mindful yoga intervention, as so far only qualitative assessments in adults of higher ages were previously done. Regarding negative effects, the number of participants reporting negative effects (65.5%) is somewhat lower that a study in young adults from the general population following mindfulness training (87%; Aizik‐Reebs et al., 2021). This might indicate that when added to treatment as usual, a mindful yoga intervention leads to fewer negative effects, or that in female populations it leads to fewer negative effects than in mixed sex populations. In addition, this study provides new knowledge about the range of negative experiences young adult women with MDD can have during a mindful yoga intervention.

In our RCT (Vollbehr et al., 2022), we did not find a significant difference between the yoga group and the control group regarding symptoms of depression, quality of life, better daily functioning, rumination and dispositional mindfulness. Body awareness and self‐compassion were the only variables that showed a group difference in the RCT, which are also positive effects reported in the qualitative interviews. However, the range of positive experiences with mindful yoga was much broader than this. This might mean that the questionnaires that we used in the RCT did not accurately capture the positive effects experienced by participants such as positive affect, feelings of relaxation, meta‐awareness, acceptance and making better choices regarding self‐care. Another possibility is that the questionnaires in the RCT only captured a specific amount of time, for instance the past week (Depression, Anxiety and Stress Scales) or the past 3 days (Hamilton Depression Rating Scale), and that this amount of time was not enough to actually capture the change during the mindful yoga intervention. However, other questionnaires did not use a time window, but assessed statements in general (Multidimensional Assessment of Interoceptive Awareness, Five Facet Mindfulness Questionnaire, Self‐Compassion Scale), which might plead against this possible reason for finding more positive effects with our qualitative measures. However, two of these latter variables (body awareness and self‐compassion) did show change in the RCT.

Another possibility is the idea that it is the other way around: The mindful yoga intervention was a positive experience for most, but did not work as a treatment for depression, as the intervention was intended to do. In line with this latter idea, it is interesting to note that we found no statements that fell within the category of *Depression, Dysphoria or Grief* of the Lindahl et al. codebook (Lindahl et al., 2017). None of the participants reported a direct effect of decreased (or increased) depression. This might indicate that the mindful yoga intervention did not directly work on decreasing symptoms of depression.

To follow another potential explanation of our findings, researchers have recently drawn attention to the idea that RCTs, with their focus on group differences, might obscure individual improvement or deterioration (see for instance Slofstra et al., 2019). For example, an individual patient data meta‐analysis found depressed individuals following cognitive behavioural therapy to differ in the amount of improvement they experienced and found a number of individuals (13%) to deteriorate (Vittengl et al., 2016). Using our qualitative assessments, we were able to look beyond the analyses of means in our RCT, to individual experiences with mindful yoga. In addition, the focus on improvement of symptoms of depression in the RCT might have concealed improvements in other domains that could have had a positive effect on (dealing with) depression. Positive affect, general physical relaxation, meta‐cognition, acceptance, change in effort or striving in daily life, mental stillness and breathing changes were not included in outcome measures of our RCT, yet were most reported as positive effects.

Regarding negative effects, our finding that most participants also experienced negative effects of the intervention is not in line with findings from other yoga RCTs, as they generally report very low numbers of adverse events related to the intervention (2.2%, Cramer et al., 2015). A review of epidemiological studies on the incidence proportion of an adverse event in a yoga class was 22.6% (Cramer et al., 2017), still much lower than the number we found. Most adverse events were issues to the musculoskeletal system (e.g., sprains and strains); digestive, neurological or respiratory adverse events were also reported but not as frequent (Cramer et al., 2017). In this study, we found the range of negative experiences to be much broader, including meta‐cognition, agitation or irritability, inflexibility, pain and self‐conscious emotions. This difference might be due to different assessments of adverse events. Most RCTs and surveys use one standardized question to assess adverse effects in an (online) questionnaire that is answered by selecting yes/no or typing the adverse event in the response section (Cramer et al., 2017). In our qualitative interview, we used several open‐ended questions and sub‐questions to assess the nature of the adverse effects. Even though the definition of adverse events might be comparable between most RCTs and our qualitative study (i.e., ‘any undesirable experience during the course of the study’ [Cramer et al., 2015] versus ‘an experience that was “unexpected, challenging, or difficult”’ [our study]), the qualitative assessment might have given a much broader overview of the range of experiences during a mindful yoga intervention than was found in (online) questionnaires.

Meta‐cognition was most reported as a negative effect. In addition, other experiences labelled as negative were noticing unpleasant emotions or physical states. This is in line with the purpose of a mindful yoga intervention, which is developing more mindfulness. Our findings in young women with MDD might echo the findings in the general population when it comes to increased affectivity and monitoring of present moment experience (Aizik‐Reebs et al., 2021; Baer et al., 2021; Binda et al., 2022). This is also suggested by Davidson and Kaszniak (2015): When individuals become more aware of internal experiences, they might become more dysphoric, as they notice things that are difficult (such as negative thoughts, difficult feelings). This is also in line with the Monitor and Acceptance Theory (Lindsay & Creswell, 2017), which states that mindfulness interventions might enhance awareness of one's experiences which could lead to increased affective reactivity. Next, acceptance is necessary to reduce the affective reactivity; however, generally these acceptance skills require more time to develop (Baer et al., 2012).

Even though these experiences might be temporary and transient when individuals develop additional skills, initial negative experiences could have been reasons for dropping out—that is, participants experiencing only negative effects or no effects were more likely to discontinue the intervention. When individuals drop out of a mindful yoga intervention, they might miss out on the time that is required to learn acceptance and skills to deal with the increased awareness and affective reactivity. In our mindful yoga intervention, participants were instructed about what they could expect, including becoming more aware of unpleasant thoughts and feelings, as well as encouraged to develop a more accepting attitude towards these experiences (Vollbehr et al., 2021). We used an online module with psycho‐educational information regarding potential effects of yoga interventions, normalizing these experiences and offering coping skills such as self‐compassion and an attitude of self‐care and acceptance. However, this module was not used very frequently by participants (Vollbehr et al., 2022), and therefore, they might have missed this additional information that could have helped them with the increased monitoring of difficult experiences. In addition to instructions in the group sessions and online module, it might be important in mindful yoga interventions for individuals with MDD to offer counselling regarding (initial) effects of becoming more mindful of present‐moment experiences and development of acceptance skills to reduce potential drop‐out.

### Rigour of the data, strengths and limitations

In order to assess the rigour of the data, it is important to consider several aspects (Tracy, 2010). First, regarding the amount of data to support our findings, we were able to collect interviews from a large percentage of participants (66%), which is higher than one of the two qualitative studies alongside an RCT (44%, Kinser, Bourguignon, Taylor, & Steeves, 2013), but lower than the other study (79%, Uebelacker, Kraines, et al., 2017). We believe that this high number indicates that we included a representative part of the total sample of participants of the mindful yoga intervention. In addition, our quantitative analyses of demographic and clinical variables of participants that did and did not complete an interview indicated no significant differences between the two groups (except for amount of yoga practice at post‐intervention), supporting the representative sample that we included. Second, our analysis was based on the Lindahl et al. (2017) codebook, but adapted in order to meet participants' experiences. This contributed to the rigour of the data, showing our dedication to accurately capture the phenomenology of these experiences. As our findings show, we were able to capture a large range of positive and negative experiences. Third, the transparency about our data analytic process further contributes to the rigour of our findings. In addition, the use of the structured coding model derived from Lindahl et al. (2017) and the changes we made and report in this paper allow a careful and structured analysis of experiences that is transparent and comparable with other studies using the same coding method.

A first limitation of this study is its generalizability. As only young women were included, our findings cannot be generalized beyond this group. It might be an interesting question to address whether men have similar or different experiences with mindful yoga than women. Second, another potential limitation may be that we asked participants about their experiences quite long after the intervention (after the 12‐month follow‐up). We did this to not interfere with the quantitative assessments of the RCT. However, this long timeline might have limited the accuracy of recall of experiences. Third, even though our sample was representative of the total sample of participants of mindful yoga, we did have a percentage of participants without a qualitative interview (33%), which might have limited a complete overview of potential negative and positive experiences of all participants. Fourth, the first author (N.K.V.) had several roles in this research, including being the yoga teacher, supervising the research assistant and writing this manuscript. Even though we tried to separate different parts of the research (e.g., N.K.V. was not involved in the data collection), we cannot rule out that this somehow has influenced the results. Fifth, given that this is a qualitative study, we cannot guarantee that the coding by the raters was free from their own experiences and biases. We hope to have minimized this by having the third rater (N.K.V.) included in the discussions and by having the other co‐authors included as ‘critical friends’ during the process of coding and presenting the data.

### Recommendations from research and clinical practice

The findings from our qualitative study provide some ideas that may be important for future studies. First, we recommend including structurally a qualitative assessment of positive and negative effects alongside an RCT as this will give a much broader overview of these experiences. We also recommend assessing broader domains of outcome measures in mindful yoga RCTs than symptoms of depression only, potentially including positive affect, meta‐cognition, general physical relaxation, body awareness, acceptance, change in effort or striving in daily life, mental stillness, breathing change and self‐compassion. As a recommendation for clinical practice, we believe it is important for clinicians to inform potential participants of a mindful yoga intervention about the range of experiences they can have during the intervention. This includes discussing experiences that are beyond a decrease of symptoms, which might be important for young adult women with MDD, in order to fully understand their learning processes. In addition, we stress the importance of informing participants what to expect from a mindful yoga intervention when it comes to potential negative experiences. A related suggestion is to offer counselling during the initial phase of the intervention, as this might be helpful in reducing the number of drop‐out due to challenging experiences.

## AUTHOR CONTRIBUTIONS

**Nina K. Vollbehr:** Conceptualization; methodology; formal analysis; writing – original draft; project administration. **Brian D. Ostafin:** Methodology; writing – review and editing; supervision; funding acquisition. **Agna A. Bartels‐Velthuis:** Conceptualization; methodology; investigation; supervision; writing – review and editing. **H. J. Rogier Hoenders:** Methodology; supervision; funding acquisition; writing – review and editing.

## CONFLICT OF INTEREST STATEMENT

The authors declare no conflicts of interest.

## ETHICS STATEMENT

The authors assert that all procedures contributing to this work comply with the ethical standards of the relevant national and institutional committees on human experimentation. The study was conducted in accordance with the principles of the Declaration of Helsinki (version 2013) and the Medical Research involving Human Subjects Act (in Dutch: WMO). All procedures involving human patients were approved by the Medical Ethical Committee of the University Medical Center Groningen (registration number NL.59324.042.16/2016/533). Informed consent was obtained from all participants before inclusion in the study.

## Supporting information


Appendix S1

Appendix S2

Appendix S3

## Data Availability

The data that support the findings of this study are available on request from the corresponding author, NKV.
