# Supplementary material for: Exploring young women's experiences of a mindful yoga intervention for depression in the Netherlands: Qualitative analysis of positive and negative effects
Source: Br J Clin Psychol. 2025 Sep 17;65(1):180–98. doi: 10.1111/bjc.70013 (PMC12889213; doi:10.1111/bjc.70013)
Supplement: Supplementary file 1 — Appendix S1 [file BJC-65-180-s003.pdf]

Affective Domain	Description	Inclusion Criteria	Exclusion Criteria
Agitation or Irritability	An agitated or irritable mood, possibly accompanied by restlessness, distractibility or uneasiness.	Agitation and irritability are types of affective reactivity and may include descriptions of restlessness, distractibility, or uneasiness. Agitation and restlessness described with somatic language would still be coded here if an affective dimension is mentioned.	Excitation or a restless mind not attributed to yoga practice. Distinguish from Rage, Anger, or Aggression, which is higher in intensity and degree and more likely to be associated with behavioral change, though these may be coded in proximity if agitation and irritability develop into anger, for instance.
Crying or Laughing	Crying and laughing, and associated vocalizations.	Crying or laughing - for any reason or none, associated with emotional content or not, and either during formal yoga or not - that is causally attributed to yoga. Include descriptions of associated vocalizations such wailing, moaning or others. May be double-coded with Positive Affect.	Crying or laughing that is not attributed (in whole or in part) to yoga practice. For instance, crying or laughing due to an environmental stimulus or social situation that is not impacted in some way by the effects of yoga.
Positive Affect	A state of positive or elevated mood or energy level, ranging on a continuum from low to high arousal.	Positive affect attributed to yoga practice. Possible descriptions of positive feelings ranging from low to high levels of arousal include: peace, rest (or a feeling of being rested), vitality, joy, love, gratitude, happiness, awe, wonder, excitation, enthusiasm, effusiveness, bliss, euphoria, ecstasy, rapture, grandeur, grandiosity, mania, or others.	Positive affect stimulated by external circumstances without clear association with yoga practice. General references to an experience being positive that don't clearly reference an elevated mood or other criteria denoting an affective change.
Rage, Anger, or Aggression	Feelings of intense displeasure or a retaliatory response, often caused by some adverse stimulus provoking an uncomfortable emotion.	Extreme feelings of displeasure, retaliation, anger or aggression, either in reaction to a stimulus or in the absence of a known specific stimulus. Rage, anger, and aggression may arise on their own as primary phenomenology, or they may be in response to yoga-related experiences or how such experiences were managed. May be double-coded with or coded in proximity to Agitation or Irritability.	Feelings of anger arising outside of a yoga-related context, in the past (before onset), or for reasons not causally linked to yoga. Differentiate Agitation or Irritability, which is marked primarily by reactivity, from Rage, Anger or Aggression, which is higher in

			intensity and degree and has different behavioral manifestations.
Re-experiencing of Traumatic Memories or Affect Without Recollection	Either a recollection of some past traumatic event in the subject's life that may or may not have been repressed, and which is generally associated with strong emotions, or the upwelling of strong emotions without any corresponding memory, content, thought or other identifiable stimulus.	Re-experiencing of traumatic memories or traumatic flashbacks include references to the recollection of some past traumatic event in the subject's life. These can also be coupled with the explicit mention of its relation to powerful emotional content such as grief, terror, or shame, in which case double-coding for those may also be warranted. Affect without recollection includes references to an experience of an unexpected, or sudden onset or upwelling of emotions without an identifiable stimulus or typical causal factor, such as a memory, a thought, or an evocative external circumstance.	Reference to some past event in the subject's life but without mention of it being emotionally intense or traumatic. Reference to emotions or affective response which have clear causal relationship to thoughts, memories, or external circumstances and are not unexpected. Reexperiencing of trauma or emotion not attributed to yoga practices and/or arising in non-practice contexts would also be excluded.
Self-Conscious Emotions	Emotions relating to one's sense of self and identity, as well as the awareness of reactions of others to oneself, whether real or imagined.	Include descriptions of notable increases or decreases in self-conscious emotions (guilt, shame, embarrassment, envy or pride) caused or intensified by yoga practice. Code self-conscious emotions arising as primary phenomenology, as responses to other specific yoga -related experiences, or as responses to how those experiences were responded to.	Self-conscious emotions arising prior to yoga or in a context in no way related to yoga -related experiences should not be coded.
**Cognitive Domain**	**Description**	**Inclusion Criteria**	**Exclusion Criteria**
Change in Executive Functioning	Either an inability to perform cognitive functions of decision making, concentration, and memory that the person used to be able to perform, or an enhanced ability in these domains of executive functioning.	A noticeable increase or decrease in any capacity to think, make decisions, memory recall, or the performance of other cognitive tasks (such as concentration). Such changes may have been noted by the subject themselves or by another observer (such as family, friends, coworkers, etc.). Diminished capacities in executive functioning also co-occur with extreme forms of Mental Stillness.	Any symptomology related to emotions, such as Depression, would not belong in this category, unless it also affected cognitive abilities, in which case double-coding would be appropriate.

Mental Stillness	A state in which there are few identifiable thoughts, a perceived absence of thought, or a poor awareness about the thinking process in general.	Mention of an absence of thought, whether positively or negatively valenced, intentionally sought or involuntarily experienced. Includes both target states of "calm abiding," or "mental quiescence" associated with concentration practice, as well as unexpected, prolonged, or undesirable states of "spacing out without mind wandering," "fogginess," or a general obscuration of cognitive processes. Lack of access to thought (inability to generate thought) is an extreme and often involuntary version of Mental Stillness.	In cases of mental fog, attempt to differentiate absence or thought from a Change in Executive Functioning.
Meta-Cognition	Meta-cognition, or meta-awareness, refers to an explicit knowledge of the content of thoughts or the thinking process. Meta-cognition can also entail a higher-order cognition of processes in other domains of experience, such affective, perceptual, somatic or sense of self.	Sustained meta-cognition or meta-awareness resulting from the practice of yoga. Includes reports of a "distancing" from thoughts, seeing thoughts as "just thoughts," or as seeing thoughts as transient events in the mind. Also includes the explicit reference of a "monitoring" awareness witnessing transient somatic, affective (i.e., feelings, emotions), or perceptual events.	A brief moment of meta-cognition, or meta-cognition not related to yoga or to particular views and values associated with yoga.
**Conative Domain**	**Description**	**Inclusion Criteria**	**Exclusion Criteria**
Change in Effort or Striving in daily life	The degree or intensity of attempts active at pursuing something valued-as-good or as a means to a valued end. Effort may be mental, physical, or emotional.	An increase in the effort, striving, trying, diligence or pursuit of a goal in daily life. Includes reports of zeal, enthusiasm, or wanting strive or put effort into a certain goal for an increasingly long duration or increased frequency. Conversely, a diminishment in effort, striving, trying, or active pursuing of a certain goal in daily life. Includes reports of a decrease in persisting demands	Increase or decrease in effort, striving, drives, or the pursuit of goals related to yoga practice. These should be coded under the original category Change in Effort or Striving.

		from daily life and an increase in taking some time off from demands from daily life.	
Change in Motivation or Goal in daily life	The reasons, drives, and needs behind a practitioner's actions, which influence or determine their behavior, as well as their expectations concerning a particular behavior.	Includes references to changes in the effect of or influence of motivating factors or goals on behavior, which may be double-coded with Change in Effort, as well as to references in the change of the motivating factors or goals themselves, which may be double-coded with Change in Worldview. Includes references to the appearance of newly desired expectations about the results of a particular new behavior. Includes changes in reasons for taking up a particular new behavior, for changing practices, and for continuing to perform a particular new behavior in the face of challenging or difficult experiences.	References made to yoga-related behaviors should be coded in the original category of Change in Motivation or Goal.
**Somatic Domain**	**Description**	**Inclusion Criteria**	**Exclusion Criteria**
Breathing Changes	Altered respiration rates that may manifest as a temporary cessation, or speeding up or slowing down of breathing.	Breathing irregularity, either during formal yoga or not, that is attributed to yoga practice. Pre-existing breathing irregularity made worse by yoga practice. Can also include symptoms like labored breathing or a feeling of suffocating. Breathing irregularity may be valenced as positive (calming, relaxing, feeling that it is easier to breathe) or negatively valenced (distressing, feeling of suffocating, etc.).	Breathing irregularity not attributed to yoga practice. Pre-existing breathing irregularity not made worse by yoga practice. Intentionally controlling or regulating the breathing should not be coded.
Cardiac Changes	Irregular heartbeat, heart palpitations, or other significant irregularities. Or the opposite, a more regular heartbeat, and less cardiac irregularities.	Cardiac irregularity attributed to yoga practice or pre-existing cardiac irregularity made worse by yoga practice. Cardiac changes include tachycardia (unusually rapid heartbeat, even at rest), brachycardia (unusually slow heart beat), and heart palpitations. Includes references to the opposite, a more regular heartbeat, and less cardiac irregularities.	Cardiac irregularity or less irregularity not attributed to yoga practice; pre-existing cardiac irregularity not made worse by yoga practice.

Fatigue or Weakness	A feeling of exhaustion, fatigue or weakness (general or localized).	Feelings of exhaustion, fatigue or weakness (general or localized) attributed to yoga practice. Also includes (but not limited to) statements about chronic fatigue, which may co-occur with increased sleep need (and therefore should be double-coded with Sleep Changes). Fatigue may also correlated with cognitive impairments such as Change in Executive Functioning.	Pre-existing conditions that involve fatigue, or the development of fatigue that is attributed to a source other than yoga (such as Lyme Disease or other medical history).
Headaches or Head Pressure	Ache, sharp pain, or pressure in the region of the head or neck. Or the opposite, a diminishing or decrease of headaches or head pressure.	Headaches or head pressure attributed to or exacerbated by yoga. Headaches includes migraines or head pressure often associated with breathing. Includes any brief, prolonged, or intermittent sensations of pressure in the head as well as any associated or subsequent pain or discomfort. Includes references to the opposite, a diminishing or decrease of headaches or head pressure.	Headaches or head pressure not attributed to or exacerbated by yoga practice, such as from head trauma or a sinus headache. Pain elsewhere in the body should be coded accordingly with the general category.
Inflexibility	Experiences of being inflexible or stiff.	Experiences of being inflexible or stiff. Not being able to perform certain yoga postures or practices due to this inflexibility or due to other physical limitations.	References to pain or soar muscles need to be coded under Pain. References to experiences of being “confronted with” or “becoming aware of” inflexiblity need to be coded under Meta-Cognition.
General Physical Relaxation	A general state of bodily (physical) relaxation	A general state of bodily (physical) relaxation, attributed to yoga practice.	General physical relaxation, not attributed to yoga practice. Head Pressure connected to breathing is coded in its own category. A release of pressure or tension in specific areas of the body should be coded under Pressure, Tension or Release of Pressure, Tension.

Pain	Pain is an unpleasant physical sensation, either diffuse or acute, and lasting for variable amounts of time. Or the opposite, a diminishing or decrease of pain.	Pain of any kind attributed to yoga practice beyond typical and expected postural aches and pain. Include also the relieving of pain attributed to yoga. When pain or the alleviation of pain is mentioned in conjunction with Pressure, Tension or Release of Pressure, Tension double-code these two together. Includes references to the opposite, a diminishing or decrease of pain.	Pain not attributed to yoga practice. Expected or anticipated levels of postural pain arising on account of the somatic immobility entailed in the practice of yoga and that is casually mentioned without attribution of significance or downstream consequences. Pain in the head and neck area should be coded under Headaches.
Sleep Changes	Changes in sleep amount, sleep need, or sleep depth.	Changes in sleep amount or depth (including intensification of existing symptoms) attributed to yoga practice. Includes (but not limited to): (diminished, decreased or increased) difficulty falling asleep or staying asleep through the night, insomnia, decreased or increased sleep need, experiencing more sound of better sleep, hypersomnia (excessive daytime sleepiness or prolonged nighttime sleep), or other related symptoms.	Changes is sleep amount or depth not attributed to yoga practice.
**Yoga skills Domain**	**Description**	**Inclusion Criteria**	**Exclusion Criteria**
Acceptance	Increased acceptance of emotions, feelings, thoughts and other experiences.	Reports of an increase of accepting emotions, moods, feelings, thoughts, habits or other experiences. Includes references to more self-acceptance. Being able to accept certain situations of events (present or past) can also be coded here. Examples are “no need to change”, “no need to fight against it”, “no need to be perfect”.	Report of an increase of acceptance, not related to yoga practice.
Body awareness	Increased sensitivity to bodily signals and adequate responding to these signals.	Being more sensitive to bodily sensations (internal stimuli). Includes references to being more receptive to bodily signals, “listening to the body”, taking time to really assess the body and bodily signals. Included references to being able to respond more adequately to bodily signals.	Being extremely sensitive to sounds, light, colors, tactile sensations or other environmental stimuli should be coded as Perceptual Hypersensitivity.

Mindfulness	Increased awareness of and presence in the moment	Reports of an increased awareness of or presence in the moment. Includes references to being in the "here-and-now", doing daily tasks (i.e., brushing teeth, drinking tea, walking) with more awareness, being less distracted with to-do lists and any references to being less in the "mode of the automatic pilot".	Becoming more aware of distracting thoughts needs to be coded under Meta-Cognition. Becoming more aware of bodily signals and bodily experienced need to be coded under Body Awareness.
Self-compassion	Increased self-compassion or decreased self-criticism.	Reports of an increase in self-compassion, self-love or self-care, or a decrease of self-criticism, self-judgement or self-loathing. Includes reference to being more "kind" to oneself, being less "hard" or being "gentler", "taking better care of themselves", and being less judgemental.	Changes in behavior regarding a specific goal should be coded under Change in Effort or Striving in Daily Life.

Note. Writing in blue are changes and additions of the original phenomenological codebook of Lindahl et al. (2017; [1]) made for our study.

References

1. Lindahl JR, Fisher NE, Cooper DJ, Rosen RK, Britton WB. The varieties of contemplative experience: A mixed-methods study of meditation-related challenges in Western Buddhists. *Plos One*. 2017;12(5):e0176239.
