# Supplementary material for: Exploring young women's experiences of a mindful yoga intervention for depression in the Netherlands: Qualitative analysis of positive and negative effects
Source: Br J Clin Psychol. 2025 Sep 17;65(1):180–98. doi: 10.1111/bjc.70013 (PMC12889213; doi:10.1111/bjc.70013)
Supplement: Supplementary file 2 — Appendix S2 [file BJC-65-180-s002.pdf]

**Electronic Supplementary Material 2**

Positive effects – participants' statements

| **Domain** Subcategory | # | % | **Statements** |
|---|---|---|---|
| **Affective Domain** Positive Affect | 33 | 56.90 | "[I became] calmer."<br>"It [the yoga intervention] calms you down a bit."<br>"I always felt better when I left [the yoga class] than when I came. [The yoga practice] always gave me a sense of productivity. And I've always felt that productivity is a great weapon for me against my depression. Not that [productivity] can solve everything, it can be a very good positive influence. So, that's why I've always walked away from [the yoga intervention] very positively."<br>"I liked it and [it was] very nice to have a moment of peace."<br>"After [the yoga] I am really calmer."<br>"Breathing exercises made you feel calmer […]."<br>"[The effects were] that you become calmer. And less anxious."<br>"[I noticed] that I came home [after the yoga class] much calmer […]."<br>"I have done many [yoga] exercises at home because I didn't feel so good, and that helped me. It gave me support in these days [of the yoga intervention]. If [I felt] really bad then yoga helped me get out of it."<br>"[I noticed] that certain exercises calmed me down."<br>"[I've learned] to really calm down for a moment."<br>"I found it […] very calming."<br>"I felt recharged after every [yoga] class."<br>"[The yoga class] felt very nice and calming."<br>"Each time I went [to the yoga class I felt] calmer and recharged […], more energized."<br>"I noticed […] when I went to yoga class […] that I really benefited from it. [The class was] calming me down."<br>"[I've learned] that it's really nice to take […] a quarter of an hour and sit calmly."<br>"I noticed that I just liked it [the yoga intervention] and it was nice. [...]. Maybe sometimes in the morning [I felt] something like 'actually [I don't] feel like doing [yoga]', but if I [did it] then I felt really nice."<br>"I was always very tired after the training. It made me totally zen. If we had to lie down at the end, I had to do my best to stay awake, it made me so calm. Delightfully calm."<br>"I always found [the intervention] very calming."<br>"I just calmed down. I came home calmer than when I went there [the yoga class]." |

| | | | |
|---|---|---|---|
| | | | “[The effect was] that you notice in some positions that you became emotional. If there was a certain twist in my body then […] something came loose. Then you became a little sad, but that was positive, even though it was negative at first.”<br>“Unfortunately, I'm not doing better now. But I do notice in myself that when I [practice yoga] I feel much calmer and that I feel better.”<br>“You always left [the yoga intervention] very calmly and it felt very nice.”<br>“I don't know if [the yoga intervention] made me less depressed, but I did feel that, especially after [the yoga class], I was calm.”<br>“It is striking that I sometimes felt calm [during the yoga class] because this is an exception in my life.”<br>“When I think about it now, I think it [the yoga practice] did help me in the way I think about certain things in order to calm down.”<br>“The yoga turned out […] to be much more calming in the moment than I first expected.”<br>“I did notice that after [the yoga class] I felt very calm.”<br>“I felt that [the yoga intervention] made me very calm.”<br>“After training I often feel really good; nice and peaceful.”<br>“[I learned in the intervention] a lot to [help me] calm down.”<br>“[It] [the yoga class] gave me a good feeling. That I had done something, which made my day a bit brighter.”<br>“What I especially noticed was that I was calm during and after the session, which was very striking for me because I was always tense and [with my awareness] in my head.”<br>“I have become calmer [because of the intervention].”<br>“I do notice that I really liked [the yoga intervention]. That it calmed me down.”<br>“[I] came back [from the yoga class] very calm. Completely balanced.”<br>“When you left [the yoga session], you [had] already closed the day. When I came out of there in the evening it was a kind of closure of the day. Instead of having to wait until you can go to bed. I just loved doing that [yoga] at night. No need to think. I think it did me good.”<br>“[I noticed] that [the yoga intervention] made me a lot calmer.”<br>“I notice that I find a lot of peace in [the intervention].”<br>“All the worries or stress of the day just go away [when I do yoga]. If something bothers me now, for example sometimes negative thoughts, which everyone has, I now think: 'oh, wait that doesn't help'. And then I go […] do [yoga] exercises at home. That helps. Then I just feel good again.”<br>“[The yoga intervention] has calmed me down. The training made me calmer.”<br>“[In the intervention I noticed that I was] physically rested as well as mentally rested. After you have just finished the training, you feel rested.”<br>“[The yoga intervention] certainly contributed to my recovery. Because you really have a moment to yourself. Then you are calm and you can put things better into perspective.” |

| | | | |
|---|---|---|---|
| | | | “You felt less rushed [during the yoga class]. I felt more like ‘it will come, no need to rush’. During training, I sometimes fell nearly asleep. It was so calming […].”<br>“I have found more peace [because of the intervention].”<br>“[The yoga intervention] was very nice and very soothing. In fact, it was so soothing that I fell asleep twice. I just really connected with myself.”<br>“I have to say that I am a bit calmer.”<br>“I've learned that exercise is good for you. When I got out of there [the yoga class] I always felt better than when I came.”<br>“Sometimes when I can't sleep or I need to calm down, I do those breathing exercises. To take some rest. Then I calm down again. [And …] I fall asleep more easily afterwards.<br>“I think overall [I] have calmed down more. [I feel] less rushed.” |
| **Cognitive Domain** Meta-Cognition | 26 | 44.83 | “[You became] a little more aware of yourself.”<br>“[I learned to] feel like: ‘I can take some time for myself'.”<br>“[I've learned] that it's nice to [work not for others but] for yourself.”<br>“[I noticed that it makes you] more aware of your goals later.”<br>“What I always do now is to pay attention to my breathing, and then [use that to] come back [to with my attention].”<br>“[I've noticed that] being in motion helps. [It helps to] get out of your mind. That you're busy [doing yoga exercises], not thinking so much anymore. So, the [negative] feeling decreases and everything seems less bad.”<br>“I was worrying a lot at the time. I was in my head a lot. So that was a mental challenge. With yoga I learned to apply that [bring my awareness out of my head, do a body scan] and that made me stop [worrying all the time].”<br>“Then I thought [...] 'don't you all be plain Janes, talk a bit' [to other participants in the group], and [...] I thought [in the group I] am the party maker. This was not helpful at all. Not that it bothered others, but it wasn't helpful to myself. Then I started to adapt. This was a mirror to me. I was caught up with [everyone in] the group. Then I ended up taking a lesson out of this: It takes energy to be caught up with that group and I'm not going to get anything out of this. So, it is better [for me] to follow the yoga class instead of being caught up with others. And not to feel the responsibility for the group.”<br>“I never really […] noticed that I was also breathing faster. Now I notice that if I pay attention to that [fast breathing] and bring my awareness there for a while, it becomes a bit better.”<br>“[I've learned] to be more aware of myself; of sounds around me and the sun shining […].”<br>“[I've learned] that sometimes I can be more [there] for myself. It gave some insights about myself; how I react to certain things, which habitual reactions or emotions I have in a certain situation.”<br>“[I noticed] that you became a little more aware of your breathing.”<br>“[The yoga intervention] has made me more aware of my thoughts and that's very important when you have depression.”<br>“I think mainly the awareness [is what I learned in the yoga intervention].” |

| | | | |
|---|---|---|---|
| | | | “[I was] really pleased that you […] learned more about your feelings […] [because of the intervention]. That you give something a moment's thought.”<br>“[I've learned] to take a moment to think about what you're doing and what you're occupied with. [Notice] your thoughts. And also, if something nice or something not so nice happens, you start thinking about what exactly happened and how you feel about it. Suppose something bad happens or something nice happens, then I just think about it.”<br>“I notice that I can separate my thoughts more from myself, so that I am not my thoughts. Yoga tells you every time to 'just let those thoughts come and go'. That works very well [for me]; that you say 'oh, how funny that I'm thinking this again' instead of that is me. So, I really learned that from [the intervention].”<br>“In some positions you noticed that you became emotional. If there was a certain twist in my body then […] something came loose. Then you became a little sad. That was positive, even though it was also negative at first.”<br>“[I've learned] that when you feel something with emotions, you just stop and think, 'What is this? And where does it come from?'. [...] Thoughts that you can then disconnect from yourself. So, an example: if you are going to do something stressful, that you can disconnect from it.”<br>“That was really something that didn't happen that easily for me [before the intervention]. That I get emotional, that I can be with [my emotions with] other people. It [the yoga intervention] brings up feelings that I was hiding.”<br>“I think it [the yoga intervention] made it clear how important relaxation is. That I am more aware of that.”<br>“I occasionally have those moments where I think 'how do I actually feel?' [...] I think […] that is now in my routine. Before [the yoga intervention], I wasn’t thinking about 'how do I feel mentally?' Before [the yoga intervention] it was just keep going, keep going, keep going.”<br>“[The yoga intervention helped me realize] that you can just be there as you are. That you can give yourself a moment’s thought. If that doesn't work, that's fine. That it is allowed. That it's not failure and it does not mean you're weak […]. I try to […] take a step back. Sometimes this [taking a step back] works and other times I find this difficult. [It is about] listening to yourself.”<br>“[In the intervention I learned] to allow if something goes wrong. In exercises I always went a step further from that which I was able to. I needed to be able to endure it longer. I tried to do that differently in those weeks [of the intervention]. Why should I? Why do I have to put my head on the ground like this when my body indicates that if I am 30 cm away [from the floor] it is better? I have tried to do that. During the training you are really made aware that you can also choose something less. Do what you can and you don't have to [do everything]. [the yoga teacher] says it and then you realize it. Also, with those home practices. [The yoga teacher] had recorded that. She also says that 'now we are going to do those exercises, but you should only do what you can. You shouldn't overburden yourself. You can feel something, but it shouldn't become unpleasant'. That was very nice. This came back every time, as a reminder for me. 'Oh yes, I don't have to go to the extreme, I can do a little less. That's nothing bad. That's okay. That's okay’.” |

| | | | |
|---|---|---|---|
| | | | “I remember one time [during a yoga class] very well. There was a lot going on in my mind and I was […] running away from it. [On my way home from that yoga class], I was crying on my bike. The emotion finally came out. I could finally pause and let it out. That gave space [...]. If you let [the emotion] out, you can also do something with it instead of pushing it away.”<br>“[The yoga intervention] has given me more peace of mind. Before [the yoga intervention] I felt I was running away from [my emotions] more. Now I notice when I get a bit restless or tend to withdraw myself, [this means] I have to pay attention to myself.”<br>“I have become more aware of my body and the things I feel. I have become more aware of my feelings. When someone asks me how I'm doing, I used to tend to say ‘I'm fine’, but now I can think about it better and answer more honestly.”<br>“I am much more aware of things. I'm less of a machine, like 'yes, this is all happening to me'. I am more aware. I take more moments for myself and I listen to myself better. That yoga group helped a lot with that. I really found it very helpful even though I was not open to it [the yoga intervention] at first.”<br>“[What I've learned] is mainly a better way to talk to myself than I did before [the yoga intervention].”<br>“I noticed that if you are busy [with yoga exercises] then you cannot be mentally busy at the same time. […] I found that really helped. To have such a moment. Take a moment to focus on yourself.”<br>“[I learned] to return to physical consciousness. [I use that] when I notice that I'm wandering off, that I get a little overwhelmed. That is useful in these moments, because it can be done anywhere. I can use that anywhere and that is a nice tool.”<br>“[I learned to listen] to my breathing. […] I became more aware of that.”<br>“[I have] learned, above all, to feel in your body; with something like a body scan. Try to really feel what you feel in your body. Become aware of what you actually feel. That you feel tension in your body. I now notice much sooner when I am too tense. Or I think: '[I] have to pay attention, it's too much'. That has been the greatest added value of starting yoga for me.”<br>“What I learned from [the yoga intervention] [is that], based on [an exercise], I can better determine for myself how I am actually doing. And when to take a step back.”<br>“You have to listen to your body [during the yoga session]. Then sometimes it happens that you unexpectedly felt emotions that you don't really want to feel. Like sadness or anger. That will all come to the surface [in the yoga class]. That's not nice at the moment [it is happening]. But in the long run: It just clears you up.”<br>“[I've learned that] if I feel uncomfortable, I stop for a moment and see where the feeling comes from. Then I can often become more aware of the unpleasant feeling.”<br>“The underlying idea [of the yoga intervention] still plays a role in my life. Becoming aware and feeling signals. Staying in the here and now.<br>When I have difficult moments, I become aware of my breathing.”<br>“During the training I was learning how to become aware of everything. […] That has had an effect.”<br>“[The yoga intervention] certainly contributed to my recovery. Because you really have a moment for yourself. Then you are calm and you can put things in perspective a bit better.” |

| | | | |
|---|---|---|---|
| | | | "[You learned] to become aware of your body. To become aware of yourself."<br>"[I noticed] that [I] needed to do certain things differently. I started thinking about that. I have forgotten [to think about] myself way too many times. What am I doing to myself? I ask such questions to myself. Indeed, it can be done differently. When I participated in the yoga I really had something like: 'now I'm doing something for myself'."<br>"[I learned] self-reflection […]. You are constantly confronted with yourself [in the yoga intervention]. That self-reflection that you can do certain things differently."<br>"[There was a] lesson in which I cried really hard. I really thought, 'Well [own name], you need to take a step back. You are doing way too many crazy things and way too much'."<br>"[You became] a little more aware of yourself."<br>"In the beginning, when I started yoga, I couldn't really feel myself. [I] couldn't relax. I didn't know whether I was stressed or not. Now [after the yoga intervention] I can feel when something is bothering me. I can consciously think about it. Do something with it now [...] or just notice it. I have much more recognition for my own feelings and the things going on in my body than I had before [ the yoga intervention]."<br>"[The yoga intervention] helped me accept everything. […] Reflecting and accepting everything. Noticing 'what's going on?' and accepting what is going on. Observing instead of the opposite [reacting]."<br>"[Since the yoga intervention], I am trying to get myself to think more about, 'something's happening. It is not fun. How do you react to it and what do you do with it?' instead of immediately getting stressed and thinking: 'oh no, the world is ending'." |
| **Somatic Domain** General Physical Relaxation | 24 | 41.38 | "[I've learned] how I can let go. Then you have to relax anyway."<br>"I've had a few times where I dozed off at the end [of the intervention]. That indicates that it is relaxing."<br>"[I have learned that it's important] to teach my body to relax. [I've learned that] when I have tension in my body, if I do some stretching exercises, that has a relaxing effect."<br>"I actually found it [the yoga intervention] very nice and very relaxing."<br>"When I practice [yoga], I can relax my body somewhat."<br>"[The yoga intervention brought me] relaxation. Getting out of your mind for a while. Focusing on your body. [After that] you don't have everything so tight anymore."<br>"Breathing exercises made you more relaxed."<br>"[The intervention brought me] the conviction that yoga and breathing and things like that really contribute to stress reduction."<br>"[The yoga intervention] helped me a lot. I got depressed because I didn't really know what it meant to make space for yourself, to relax. I was very busy studying and working and yoga has given me a space for [relaxation]. It was once a week. You just went and that was fixed. Then you had to relax and you had to lie down on the floor. The fact that I had to relax made me notice 'oh wow, I can feel that way [relaxed]."<br>"[I noticed that I liked] to relax [like in yoga class]." |

| | | | |
|---|---|---|---|
| | | | “[I've] found a different way to relax [in the yoga intervention].”<br>“Thanks to yoga [I learned] you also have to have peace. I learned that having peace and relaxation is nice. I had not experienced that feeling for a long time [before the yoga intervention]. I was actually very afraid to relax. I learned at yoga that I don't have to be that [afraid].”<br>“After the training I was just completely […] tired, but different tired than I normally was. Really relaxed-tired.”<br>“With the breathing exercises I have learned that if I feel tension, I can breathe it away a bit. Then I immediately feel calmer.”<br>“[I learned] how much your attitude can do for the panic symptoms and the tension in your body you experience. That you have to hold it [a posture] and then let go. [Then] you're really going to see the difference, when you've let go.”<br>“[I found] A little more rest in […] my body [after the intervention].<br>“[The intervention] was very relaxing.”<br>“[Often the yoga class was] relaxing for a moment.”<br>“I especially found that I was very relaxed at the end of the yoga class. I normally don't take the time to relax. And with yoga, of course I do.”<br>“[The yoga intervention] was really very relaxing.”<br>“Because [in the yoga class] you relax a bit and are busy with other things. So, you kind of forget about [other things].”<br>“Often, I entered [the yoga class] very tense, but then at three quarters in I noticed that I felt more relaxation. When you are depressed, you just feel very tense. Every moment where you can let go of that is valuable. So, being free from tension for a while, that's very special for me. And that happened a number of times, three times [in the yoga class].<br>“Doing yoga […] made me feel […] relaxed.”<br>“[You learned] to completely release all tension in your body. Just lie down and do nothing. That this can be very nice.”<br>“[the yoga intervention] was […] a moment of relaxation. This gave a lot of peace […] at that moment.”<br>“I always found it difficult to fully relax the muscles. That is something that I found especially difficult to do in the beginning. It became easier after [I had taken] more [yoga] training sessions.”<br>“To think about your toes and work all the way up [your body] makes you feel a bit more relaxed.”<br>“[My body] is a bit more relaxed [since the yoga intervention].”<br>“[I noticed after the intervention] that [my body] is a bit more relaxed.”<br>“[I've learned that] when I am panicked, […] to do some yoga or breathing exercises so I'm more relaxed.”<br>“I use [yoga exercises] as a tool that I can use when necessary. The moment I notice it's getting a bit much for me. [...] I do notice that [doing yoga exercises] helps. I'm very close to my final exam at the moment, so it quickly becomes too much. Therefore, I do [yoga exercises] every couple of days now. It's nice to have that moment of rest again.”<br>“[I've] learned to relax more.” |

| | | | |
|---|---|---|---|
| | | | "[During the yoga sessions I noticed] especially that muscle tension left my body. […] [I] really noticed that the tension of my muscles went away as the time [of the session] went on."<br>"I think [I am] more relaxed in my body. Less tight […]."<br>"When I do yoga, it really relaxes me and [my] body relaxes completely."<br>"[I learned in the yoga intervention] to relax."<br>"[I noticed during the intervention] very much [a sense of] peace. Because I have ADHD, my head is always thinking ten things at a time. Therefore, it is nice to learn how to relax. That was nice, because I find it difficult to really relax."<br>"[I noticed] relaxation in [the yoga intervention]."<br>"[I noticed] that I really relaxed [in the yoga class]. I [even] had a moment when I fell asleep for a while [during the yoga class]. This means that you surrender. I find that difficult, because I am always someone who wants to keep going." |
| **Yoga Skills Domain**<br>Body Awareness | 21 | 36.21 | "[I've] listened to my own body a lot [in the yoga intervention]."<br>"You are focusing on your body and things that you otherwise would have simply ignored. [...] When you focus on those [yoga] exercises you really notice 'oh that's tense', and then you learn with those [yoga] exercises how to let that [tension] go."<br>"[I'm] more aware of [my] body."<br>"[I've learned that I] have to better listen to my body. That [my body] says a lot. Listen to your body and then notice that something is wrong."<br>"[I now apply] that I breathe consciously for a moment and consciously feel my body. And listen to that."<br>"[During the yoga class] You can focus on your body; I liked that […]."<br>"[It was] nice to do [yoga]. Because it is more physical than just [being] in your head. It focuses on your body. How do you stand? I really liked that."<br>"In the beginning, when I started yoga, I couldn't really feel myself. [I] couldn't relax. I didn't know whether I was stressed or not. Now I can also feel that something is bothering me. I can consciously think about it. Do something with it now [...]. Or just notice it. I have much more recognition for my own feelings and the things going on in my body than I had before [the yoga intervention]."<br>"[I've] learned to listen to my body some more."<br>"[…] I've learned […] to recognize [bodily] signals better."<br>"[In the intervention I] became more aware of my body. I always used to feel struck when I suddenly [felt really bad]. In the training I learned to pay attention to those signals [of feeling bad]. I noticed that there is a lot going on in my body [that is telling me that I am not feeling well]. That my neck was bothering me. That I became nauseous. More headaches and body aches."<br>"[I noticed that I] was more aware of what's going on in my body [after the yoga intervention]. And [I] process [the bodily signals] in a different way than what you do in the therapy sessions, for example."<br>"[The yoga intervention helped me to] become aware of how it feels in your body." |

| | | | |
|---|---|---|---|
| | | | “During yoga I could learn from the fact that you don't have to do anything for a while. [The yoga class] was very accessible. There were always multiple options in a posture. I learned to listen to my limits. I find that very difficult, so it was very nice to experience 'it is good to stop at a certain point or take a step back'.”<br>“I take that very much with me […]. Noticing my body's signals sooner than if you don't [pay attention to those] and notice it only when it is striking.”<br>“[I] appreciated to learn much more about [my] own body […] [because of the intervention]. You think a little more about everything [that is going on in your body].”<br>“[I liked] that you really learn to listen to your body [in the yoga intervention].”<br>“[After the yoga intervention], I felt that it helped me to feel pain in my body I wasn’t aware of before. To notice more [inner] signals. To be able to breathe towards the pain […].”<br>“I notice more things in my body [since the yoga intervention].”<br>“Listening to your body. I learned a lot from that in the yoga training.”<br>“I am quite a tense person. I am always in a tense posture. I also had some back problems and such. Then I started doing yoga and [with yoga] your mindset is very much focused on how your body reacts. Listen to your body. What happens when there is something [wrong] or when you hear something that is not so nice? How does your body react to it and what can you do about it?”<br>“I think [the yoga intervention] helped to become more aware of […] your body.”<br>“[I've learned to] listen to my body a bit more when I need to rest.”<br>“[I notice] that I really listen better to my body now [after the intervention]. And that just makes you feel better.”<br>“[I've learned to] pay attention to 'where do I feel stress? Where is that in my body? And what does that say’?”<br>“[I have learned to …] Become more aware of [my] body. That you can feel ‘how is my body? How is my body? What do I need’?”<br>“In yoga you are […] focusing on 'what is my body saying?' I really think this helped in my recovery.”<br>“I think yoga has mainly taught me to learn to listen to [my] body.”<br>“I have become more aware of my body and things I feel. I have become more aware of my feelings. When someone asks me how I'm doing, I used to tend to say ‘I'm fine’. But now I can think about it better and answer more honestly.”<br>“The movements [in the yoga class] were helpful to [connect me more] to my body […]. When you're depressed you think you can't do anything at all. [But] actually you can. That's what we did there [the yoga intervention]. Take a breath. Listen to your body. Say 'I'm just here and maybe that's enough'.”<br>“[I've mostly learned] to better listen to my body. […] I just became more aware of [my body].”<br>“[I have] learned, above all, to feel in your body; with something like a body scan. Try to really feel what you feel in your body. Become aware of what you actually feel. That you feel tension in your body. I now notice much sooner when I am too tense. Or I think: '[I] have to pay attention, it's too much'. That has been the greatest added value of starting yoga for me.” |

| | | | |
|---|---|---|---|
| | | | “[I've] better [learned] to recognize [my] body, the signals. Now I think 'it is actually bizarre that I've never felt that kind of things before'. Because then I could have hit the brakes much sooner.”<br>“[I noticed] that because of [the yoga intervention] I became more and more aware of what I feel in my body. I have become more aware of feeling signals in my body.”<br>“The underlying idea [of the yoga intervention] still plays a role in my life. Becoming aware and feeling signals. Staying in the here and now.”<br>“During the training I learned […] how to pay attention to your body. How do you do that exactly? That has had an effect.”<br>“[I learned in the yoga intervention] that you should not go beyond what your body indicates. With stretching exercises don't go too far. If you do, you have to go back a bit. I apply that now. I don't go that far beyond my limits anymore. Physical and mental limits. I won't go that far beyond those anymore.”<br>“[I learned] that you become aware of your body.”<br>“[I've learned] that you think more about your body. I'm such a control freak, who often crosses [my own] limits. Now that I listen to my body more, I give myself more peace to take a moment [to pause]. Now my body is saying: 'it doesn't go any further. Now you really need to pause.” |
| **Yoga Skills Domain**<br>Acceptance | 20 | 34.48 | “[I learned that when there is] an exercise that is not working for me because I have rheumatism. That is ok. You can do something else.”<br>“I got frustrated very quickly and [was] very punishing [on myself]. I'm working on that now [since the yoga intervention]. I'm thinking 'it's good that I'm here and I'm doing what I can; it doesn't have to be perfect'.”<br>“Just accept it as it is [is what I took from the intervention].”<br>“[I] have more acceptance for my physical complaints [since the yoga intervention].”<br>“[I liked] that you have to accept yourself somewhat. Not just for physical things. Especially mentally.”<br>“[The yoga intervention brought me] self-acceptance. That it's okay. You can do it your way.”<br>“I […learned] to focus on myself. To be able to accept myself. Including things that I'm not happy with. That's normal.”<br>“[…] [I try] to accept that [the present moment] is just there instead [of] something that always needs to be changed.”<br>“[The yoga intervention] was, from [the] beginning, [...] positive. Even if I felt not so well and that came up in an exercise then I had no problem with it. [The yoga class] was […] a very safe way to just feel that.”<br>“Every week we had a theme [in the yoga class] and sometimes [there was a homework exercise for which] you had to think about something in between [the classes]. If it was a tricky theme and something got stuck in your head, it was just there. Normally I would find that very disturbing and would worry about it. But because you are also doing physical [yoga] exercises in between [the yoga classes] and you are guided through [the yoga exercises] by [the yoga teacher], [the things that was stuck in your head] was just there. It was not difficult that a feeling or a certain thought was there. It [...] just felt okay.”<br>“[I learned to …] experience without having to do anything with it.” |

| | | | |
|---|---|---|---|
| | | | “[I learned in the weeks of the yoga intervention to] look at myself differently […]. [You] accept […] yourself.”<br>“[I've learned] that it's fine if you just notice something. You don't have to change it right away. Not all the negative things are something you have to do something about. You can just notice it and let it be.”<br>“[The yoga intervention] helped me accept everything. […] Reflecting and accepting everything. Noticing 'what's going on?' and accepting what is going on. Observing instead of the opposite [reacting].”<br>“Mainly learning to let go and accept [is what I learned in the yoga intervention]. Accepting the depression is therefore much easier. Which gives better results. That is also what I learned in therapy. But with yoga it is a bit easier to put into practice.”<br>“There was occasionally a difficult posture in [the yoga class]. In which you had to stretch a bit. If [the stretching] doesn't work, it's no big deal.”<br>“[I learned in the yoga intervention] that some sensations are just there and you don't need to do something about them.”<br>“I appreciated that I could be really sad [during a posture].”<br>“[I learned] to accept something [the pain] more. I'm not that there yet, but I think I am a little closer [to accepting the pain].”<br>“Some things [from the intervention] don't fit me. […] Another time [in the yoga class] I thought 'I'm just relaxed' and then I focus on something else. It passes me by. If it doesn't work then it doesn't work. That is easier to apply when I'm calmer.”<br>“I had one time during the training that I thought I allowed my emotions a little more.”<br>“[I've learned] that bad thoughts […] can just be there. More acceptance. That it's okay to feel certain things. That helps. That you acknowledge that it [bad thoughts] is there.”<br>“[The yoga intervention helped me realize] that you can just be there as you are. That you can give yourself a moment’s thought. If that doesn't work, that's fine. That it is allowed. That it's not failure and it does not mean you're weak […]. I try to take a step back. Sometimes this [taking a step back] works and other times I find this difficult. [It is about] listening to yourself.”<br>“[I've learned] to accept the situation as it is somewhat more. Because with yoga you are not always in a pleasant posture, but that no reaction can also be a good reaction.”<br>“[During the intervention] I noticed that I was really fighting with myself. When I was not succeeding [in a posture], I could be very bummed about it. But then I think 'it's yoga, it's okay, it's the way it is'. And I learned a lot from that.”<br>“[The intervention brought me…] acceptance. [Previously] I always ran away […] from my feelings. Now I look more at 'what is it'?”<br>“[I've learned] to be at peace with myself and to accept myself and how I feel.”<br>“[The intervention brought me] self-acceptance.”<br>“[I notice] that I panic less when something is wrong. That I think 'it's there; I can't change it anyway'.”<br>“[...] When I went skiing with my family for five days, I had to take a pause every now and then. [I had to] accept my thoughts. ‘You have them. It's not decisive for you. It's not who you are. Let them go. Breathe well. Just [take] a moment for yourself.’ That did help.”<br>“[I've learned to] accept that thoughts are there and [to] let them go.” |

| | | | |
|---|---|---|---|
| | | | “The biggest change [after the yoga intervention] is that I've learned to accept more that ‘it is what it is’. That sometimes it's okay to not feel well physically or mentally. Of course, you don’t always want that. You are very disappointed about that. But I learned more from this [the yoga intervention] that ‘it is what it is’. It's okay to have a bad day. I don't have to pretend to be better than what it is. That is really a big outcome for me, what I learned from [the yoga intervention].”<br>“It's just easier to get out of your mind [because of the yoga intervention]. You get rid of those thoughts more easily. Not to get bogged down in that [thoughts] again. Before [the yoga intervention], it became such a vicious circle. You think badly and then you think 'I shouldn't think about it' and you don't accept that something like this happens. In yoga you learn: you just have to accept and you don't always have to resist. Let it come. Everyone has thoughts sometimes and you don't have to do anything with those [thoughts].”<br>“In the beginning of the training, I found [feeling sadness or anger; emotions you don't really want to feel] difficult. You resist it. I don't want something like [these feelings] to come out. Because you always want to be strong for the outside world. [...]. [But later in the yoga intervention] if it happens again [the feelings arising], let it be there. It is fine.” |
| **Conative Domain**<br>Change in Effort or Striving in Daily Life | 20 | 34.48 | “[I have learned that] I can take time for yourself.”<br>“[I learned that you can] find a moment for yourself. That you can take a rest.”<br>“[I have learned that I am] allowed to take space to just be there. Think for once that I don't have to do anything at all and then just accept that.”<br>“Every time I think 'I need to take a little time for myself' I know yoga is the answer for that.”<br>“ [The yoga intervention] helped me a lot. I got depressed because I didn't really know what it meant to take up space for yourself. To relax. I was very busy studying and working and yoga has given me a space for [relaxation]. It was once a week. You just went and that was fixed. Then you had to relax and you had to lie down on the floor. The fact that I had to relax made me notice ‘oh wow, I can feel that way [relaxed].”<br>“[I've learned] that I'm more likely to think 'I'm going to sit down somewhere'. Even if everyone else is still standing.”<br>“I especially learned that if I can't do something the way someone else is doing it, I can do it in an adapted form.”<br>“[I have learned …] that I don't have to participate in everything with the rest.”<br>“[I've learned that] I sometimes say [to myself] 'take a rest. Time for yourself, that's also important'.”<br>“I've given myself more time to slow down. [Before the yoga intervention I] just kept going and going and going.”<br>“I have learned to choose for myself. I know […] that it works well for me. I schedule a moment for myself. [When I do this] I notice more peace. And then I realize that this is necessary.”<br>“I noticed […] that my neck was bothering me. That I became nauseous. More headaches and body aches. If I feel that [now] I take a step back. Then I think 'I [should] take care of myself a little better. Take care of myself a little more'. [When I do that] it will never be as gloomy as it was [when I was depressed].” |

| | | | |
|---|---|---|---|
| | | | “[I've learned] to take a break and notice 'how do I feel now? Do I still feel like doing this? Is it still okay?'. More often [I take] a moment to evaluate instead of just continuing [what I am doing].”<br>“[I've learned] that [it] is useful in everyday life when you're stressed to take that moment of peace.”<br>“During yoga I could learn from the fact that you don't have to do anything for a while. [The yoga class] was very accessible. There were always<br>multiple options in a posture. I learned to listen to my limits. I find that very difficult, so it was very nice to experience 'it is good to stop at a certain point or take a step back'.”<br>“[…] Taking time to relax has helped me.”<br>“[During the intervention, I learned] from a common exercise that we tend to over-stretch when we start to feel the stretch. You can always challenge yourself doing that. This also applies to emotions. You can always look beyond what's right in front of you.”<br>“If I now notice that I've done too much again, I'll take my moment of rest.”<br>“[With exercises from the intervention, I sometimes] think for a moment […] about 'where do I feel what in my body?' That I then notice 'wait, pause for a moment. I'm stressed now. I have to take care of myself for a while'.”<br>“[I've learned that] if you do something, you don't have to go to the extreme, a little less is also allowed.”<br>“I occasionally have those moments where I think 'how do I actually feel?' [...] I think […] that is now in my routine. Before [the yoga intervention], I wasn’t thinking about 'how do I feel mentally?' Before [the yoga intervention] it was just keep going, keep going, keep going.”<br>“I am very hard on myself. And I learned […] in yoga that less is also allowed.”<br>“[In the intervention I learned] to allow if something goes wrong. In exercises I always went a step further from that which I was able to. I needed to be able to endure it longer. I tried to do that differently in those weeks [of the intervention]. Why should I? Why do I have to put my head on the ground like this when my body indicates that if I am 30 cm away [from the floor] it is better? I have tried to do that. During the training you are really made aware that you can also choose something less. Do what you can and you don't have to [do everything]. [the yoga teacher] says it and then you realize it. Also, with those home practices. [The yoga teacher] had recorded that. She also says that 'now we are going to do those exercises, but you should only do what you can. You shouldn't overburden yourself. You can feel something, but it shouldn't become unpleasant'. That was very nice. This came back every time, as a reminder for me. 'Oh yes, I don't have to go to the extreme, I can do a little less. That's nothing bad. That's okay. That's okay’.”<br>“I don't go on and on all the time. I think much sooner 'I need to calm down'.”<br>“[I learned] that I seek more peace. Don't continue. I know I need to take some time for myself.”<br>“[...] When I went skiing with my family for five days, I had to take a pause every now and then. [I had to] accept my thoughts. ‘You have them. It's not decisive for you. It's not who you are. Let them go. Breathe well. Just [take] a moment for yourself.’ That did help.” |

| | | | |
|---|---|---|---|
| | | | “I am much more aware of things. I’m less of a machine, like ‘yes, this is all happening to me’. […] I take more moments for myself and I listen to myself better. The yoga group helped a lot with that. I really found it very helpful even though I was not open to [the yoga intervention] at first.”<br>“[During my burnout I] experienced a lot of body aches. I didn't know what to do about them. It made me very insecure. Because I felt a lot of sensations that made me think 'I really don't know what to do about this'. [The yoga intervention] really helped me with that, because of the relaxation. By experiencing what is happening [I] learned to notice my limits earlier. Which makes me think I'm stronger now. I know better where my limits are.”<br>“During yoga, some yoga exercises were quite challenging. How long can you stand on one leg or how much can you focus your attention? Those things were challenging. I got further than I thought. So, physically [the intervention was] especially challenging.”<br>“[I learned] that you need to think more about yourself. Self-care. That's my pitfall, because I'm always busy with someone else and always taking care of someone else. I often forget about that. [I noticed] that [I] needed to do certain things differently. I started thinking about that. I have forgotten myself way too many times. What am I doing to myself? I ask such questions to myself. Indeed, it can be done differently. When I participated in yoga, I really felt: 'now I'm doing something for myself'. I never do anything for myself, always for someone else. I thought: 'now I'm really doing something for myself' and something that I really like.”<br>“[I've learned] that you think more about your body. I'm such a control freak, who often crosses [my own] limits. Now that I listen to my body more, I give myself more peace to take a moment [to pause]. Now my body is saying: 'it doesn't go any further. Now you really need to pause.”<br>“[There was a] lesson in which I cried really hard. I really thought, 'Well [own name], you need to take a step back. You are doing way too many crazy things and way too much'.”<br>“Taking time for myself. I still find that difficult sometimes, but I've learned that [in the yoga intervention].” |
| **Cognitive Domain**<br>Mental Stillness | 12 | 20.69 | “I was a little calmer in my mind [after the intervention]. That I don't have to think as much.”<br>“I [liked] the fact that you can clear your head once a week.”<br>“I was worrying a lot at the time. I was in my head a lot. So that was a mental challenge. With yoga I learned to apply that [bring my awareness out of my head, do a body scan] and that made me stop doing it [worrying all the time].”<br>“[…] Always [when I] drove home […] I thought 'I feel nice and calm'. Just cleared my head. A little more peace in my mind […].”<br>“[I noticed that] I was a little more relaxed [after the intervention]. I was a little less busy in my mind with school and things like that. I'm always quite worried about deadlines and I noticed that after yoga it was a bit calmer in my mind; that I had my thoughts in order.”<br>“[I noticed] mostly that I was calmer, calmer in my mind. [Before the yoga intervention] it was difficult for me to do nothing and focus on something simple. But during the yoga class it was easier.”<br>“It's [...] the peace it [the yoga intervention] gave me. I am very, very busy in my mind and at that moment [of the yoga class] I could really calm down in my mind. That was very important to me, because my mind can do very strange things. It's nice to leave that behind for a while. To be busy with other things for a while.” |

| | | | |
|---|---|---|---|
| | | | " [The yoga intervention] was a moment of clearing my mind completely."<br>"[The yoga intervention] has given me more peace of mind. Before [the yoga intervention] I felt I fled from it [peace of mind] more. Now I notice when I get restless or  withdraw. [I now know that then] I have to pay attention to myself."<br>"[During the intervention I was] able to relax. […] I had a lot of worrying thoughts. Yoga allowed me to concentrate on something else and this was soothing. This allowed me to let go of my thoughts at that moment, which made me more relaxed and calmer."<br>"During the training [You noticed] the calmness in your mind. After training, I noticed every time that I was a bit tired. A different kind of fatigue than what I had the rest of the time. Now [after the yoga class] I had the feeling of 'I have achieved and done something for myself' and that feels calmer."<br>"What I liked about yoga is that you are also quiet and sit still for a while. Meditation-like things. Which makes me notice that I'm a bit calmer in my mind and that I can control [my mind]."<br>"I noticed that [the yoga intervention] made me calmer and calmed down my mind. Which is special for me [as that does not happen very often]." |
| **Somatic Domain**<br>Breathing Changes | 8 | 13.79 | "I never really thought about the fact that I was breathing faster. Now I notice that if I pay attention to that and consciously go there [my breathing] for a while, it goes a bit better."<br>"[I benefited a lot from] the breathing exercises. [...] Before that, [I] suffered from panic attacks. [...] Every now and then I just started breathing wrongly and then I would either pass out or I would get dizzy and that scared me. With the breathing exercises I learned that if I feel tension, I can breathe that away a bit and then I feel calmer right away."<br>"[I learned that] when I hyperventilated, I focused on breathing. That helps a lot."<br>"[I've learned that] when I get nervous, I watch my breathing and that calms me down. We did that quite a lot with the training sessions. Pay attention to your breathing and become aware of your breathing."<br>"[I've learned that] taking a good breath helps […] [with difficult thoughts]."<br>"Sometimes I notice that my breathing is very [fast] and then I just notice it. […] And pay more attention to my breathing. [I] try to become calmer that way."<br>"[I learned how to] breathe into the pain […]."<br>"[I've learned to watch my] breathing. I tend to breath shallowly, and that's not good for you. That's what they said there [in the yoga intervention]."<br>"I still use the breathing techniques a lot. In the beginning my breathing was quite fast and it's a lot calmer now. [There are] some things that I do automatically now that I didn't do automatically, such as calm breathing. When I feel panicked, I naturally start breathing more slowly. That's what I've been taught now."<br>"[I've learned] that I can better control [my breathing]." |
| **Yoga Skills Domain** | 7 | 12.07 | "The way of thinking [that we learned in the yoga intervention] changed me. I [became] more forgiving to myself." |

| | | | |
|---|---|---|---|
| Self-Compassion | | | "I got frustrated very quickly and [was] very punishing [on myself]. I'm working on that now. I'm thinking 'it's good that I'm here and I'm doing what I can; it doesn't have to be perfect'."<br>"Breathing exercises made you […] more compassionate towards yourself."<br>"[I've learned to] be a little nicer to myself. Take myself into account. What is and what is not good for me."<br>"The mental [part] and being kind to yourself. I think that helped me a lot."<br>"[Because of the yoga intervention] I try to be less hard on myself. Before, if I made a mistake or if something went wrong, I could get very angry. And now I think 'okay, it's done, how do we proceed'."<br>"I noticed [during the yoga classes] that I wasn't that flexible. That some things didn't work out physically. That it's okay and you shouldn't get angry with yourself if it doesn't work out. In the beginning I was not yet able to do this, but later [in the intervention] it really got better." |
| **Yoga Skills Domain** Mindfulness | 6 | 10.34 | "[The yoga intervention brought me] mindfulness. If you drink a cup of tea, do so with your full awareness. Not with everything else at the same time. Give yourself those kind of moments of peace."<br>"I try to be a bit more in the here and now in daily life […]."<br>"[…] [Since the intervention], when I notice that I am very restless, I try to concentrate on what is happening in my body and what is going on."<br>"I think [I got] a little more into the here and now [after the yoga intervention]."<br>"The underlying idea [of the yoga intervention] still plays a role in my life. Becoming aware and feeling signals. Staying in the here and now.<br>When I have difficult moments, [I am] more in the here and now and not [concerned with] what I have to do, but what is happening now. I also apply this in my daily life." |
| **Conative Domain** Change in Motivation or Goal in Daily Life | 3 | 5.17 | "I started exercising more because of [the yoga intervention]. Because I got that insight 'I have to move to feel better'."<br>"I exercise a lot more now [after the intervention]."<br>"What has improved for me is that I started doing other forms of movement and that has made me feel better for the first time in a few years. I don't know if that is directly due to the yoga, but I really learned that yoga is good." |
| **Cognitive Domain** Change in Executive Functioning | 2 | 3.45 | "[…] The movement made it easier for me to be less distracted."<br>"Concentrating, I think that is an important thing that I have learned." |

| | | | |
|---|---|---|---|
| **Somatic Domain** Cardiac Changes | 1 | 1.72 | "I often suffered from palpitations. That was calmer. It was gone for a while [after the intervention]." |
| **Affective Domain** Crying or Laughing | 1 | 1.72 | "[There was a] lesson that I cried really hard. That I really thought, 'Well [own name], you do need to take a step back. You're acting way too crazy and doing way too much'." |
| **Somatic Domain** Headaches or Head Pressure | 1 | 1.72 | "[I suffer from] tension headaches. That was not gone, but often it was not present during yoga." |
| **Somatic Domain** Pain - opposite | 1 | 1.72 | "After [the yoga class] I often had less pain." |
| **Somatic Domain** Sleep Changes | 1 | 1.72 | "[After practicing yoga] I can sleep better." |
| Other | 6 | 10.34 | "Most of all, I learned that […] I am not the only person my age who has these kinds of problems [depression]."<br>"[…] I wasn't feeling very well myself. Seeing other people who had it even worse helped me. This might sound a bit harsh, but you can see that you are not alone and that it can also get worse. Your self-pity goes away somewhat. You come in [the intervention room] and you see other people who have exactly the same or have it worse and that's nice to know."<br>"[I've learned] how to better deal with my frustrations. Some frustrations when things don't work out and how to deal with that. So, for example, if we had to do an exercise where we had to lie down and a group member who had a cold was breathing noisily, that was very annoying. How can you deal with that properly? Or with the tension that is too high? […]."<br>"I do have a posture or two where I make myself very tall or wide in a room. I sometimes have used that when I wasn't comfortable at work. I had a very dominant boss and then I tend to disappear. When I practiced it [this posture helped me] to be physically present."<br>"[Because of the yoga intervention I've learned to] stay calm. For example, my son is quite rambunctious. I taught myself to count to ten. Leave him for a while. Or get down on one knee with him and say: 'why are you acting like this?'. That I really focus on him for a moment. 'Why are you doing that like that'?" |

| | | | |
|---|---|---|---|
| | | | “[I have] mainly [learned from] the fact that [before every session] I thought 'I don't want to go there' and then every time afterwards I thought 'it was kind of chill'. I notice that I do things now because I know that doing things really is better for me than sitting on the couch all day.” |
