# Supplementary material for: Exploring young women's experiences of a mindful yoga intervention for depression in the Netherlands: Qualitative analysis of positive and negative effects
Source: Br J Clin Psychol. 2025 Sep 17;65(1):180–98. doi: 10.1111/bjc.70013 (PMC12889213; doi:10.1111/bjc.70013)
Supplement: Supplementary file 3 — Appendix S3 [file BJC-65-180-s001.pdf]

Electronic Supplementary Material 3

Negative effects – participants' statements

**Domain** Subcategory	#	%	**Statements**
**Cognitive Domain** Meta-Cognition	22	37.93	"I found [my mind] wandering very quickly. It was really hard to stay focused."<br>"During [the yoga classes] I sometimes had trouble concentrating."<br>"[During the yoga classes] you had to concentrate. [When that happens] my mind starts thinking about other things a lot and I wasn't relaxed at all."<br>"I couldn't concentrate well there. [...] I [am] very aware [...] of my surroundings. With the exercises [in the yoga class] you have to relax your stomach so that you stand firm, but then I don't follow those things [instructions] but I am more occupied with the environment and not with the posture. That's why I don't perform the exercises properly and you don't find balance […], for example."<br>"[During yoga classes I noticed] that I [...] try to hold as long as I can. Of course, this was what I did all the time when I was depressed. Just keep going and going. Even though the message in yoga was 'you don't have to do more than you can handle' [I noticed] that I myself [...] kept trying to cross [my own] limits."<br>"[During the yoga classes] I suddenly really noticed all the pain [in my body]. [...] I felt fatigue in my body. [I was] really exhausted."<br>"I could get quite emotional during yoga. You try to relax and for me that doesn't happen very often. So, when I do relax, a lot happens mentally as well. I notice all the things that break me down emotionally."<br>"For me, lying still for ten minutes was like hell. I couldn't deal with that every single time. That was what bothered me the most. [...] Ten minutes of lying still with your own thoughts without distractions can be quite tough. I spend all day distracting myself so that my mind [does not] wander to the wrong things and that I get stuck in that. If you are forced to lie still for ten minutes with your eyes closed. Then you can't distract yourself with nothing at all. So that all those issues that you are always trying to leave alone and not discuss them too much, come right back at you."<br>"[During] the third lesson, […]I snapped and I noticed 'now I'm depressed again, really depressed' I was overburdened for a long time, [have] had all kinds of worries and at that point it was too much."<br>"[During the yoga intervention I had] the realization 'I'm depressed again and I don't want to go on like this'."<br>"I found lying still confrontational. You just go into silence and then you start thinking things and these are not always the happiest or nicest of thoughts."

			“Sometimes, […] on the day [of the yoga class] I was not feeling well and during the meditation you have a moment to think about that, and that can sometimes be confrontational.”<br>“You had to do quite a lot of exercises in which you had to concentrate and let go. I had a lot of trouble with that. I couldn't let my thoughts go and I just kept ruminating in my mind and I couldn't concentrate. At that moment, I found it quite frustrating that it didn't work for me. That's a bit of the problem with me anyway. I'm very perfectionistic. So, I found it very difficult that I couldn't concentrate or relax in certain exercises.”<br>“Doing yoga exercises […], something I've never done before, was quite out of my comfort zone. At that moment I was just a little too depressed to open myself up to that. I started to dread it.”<br>“Sometimes when you started with a kind of meditation beforehand [in the yoga class] you were allowed to think a bit. Every now and then I would get stuck in [those thoughts] and take that into the yoga session. I became emotional. I keep it to myself [when that happens], but that was something that [wasn’t easy] for me; That I get emotional, that I can be with the other people [with my emotions]. [the yoga class] brings up feelings that I was actually hiding.”<br>“I wasn't calm enough to participate.”<br>“Going [to the yoga training was difficult for me]. [...] I also suffered from anxiety and social anxiety. So, if I [...] had to find a certain kind of peace with a group of people around me, I was very much [getting] in my own way. I was only concerned with who was around me.”<br>“[During the yoga intervention I noticed] that I thought quite negatively about myself.”<br>“[It was difficult for me to] really learn to listen to my own limitations. I find that very difficult.”<br>“Because I have misophonia. I [have] trouble concentrating. So, [the yoga intervention] didn't really help me.”<br>“Because [when] you do yoga, they also expect you to breathe a little heavier, I think. You hear everyone breathing and I can't stand that.”<br>“At first [I found it] very difficult [...] to focus my attention. I had trouble with that. That you should focus on your breathing or on your feet. That it doesn't matter if your mind wanders, but that as soon as you realize it, you can bring your mind back. That [mind wandering] happened a lot to me at first, but I think that's normal, but I really had it [mind wandering] a lot at first.”<br>“When I'm in a room with people where everyone is dead quiet, I'm completely thinking that I shouldn't breathe too loudly. Then that’s all I can think of.”<br>“My mind was full. Therefore, I couldn't relax during the meditation. It didn't work, even though I wanted it to work.”<br>“Maybe yoga isn't quite my thing. I can't quite calm down my mind.”<br>“You have to listen to your body [during the yoga class] and then sometimes it happens that you unexpectedly felt emotions that you don't really want to feel. Like sadness or anger. That will all come to the surface. That's not fun at the moment. But in the long run: It just clears up. [...] In the beginning of the training I found that difficult. Then you resist it. I don't want something like

			that [emotions] coming out. Because you always want to be strong for the outside world. [...] [But later] if it happens again, let it be there. It does not matter."<br>"Another experience [during the yoga intervention] was that I had to try to focus on something. I am someone who is very easily distracted and was occupied with all sorts of things. I really had to get used to that. I did not succeed in [concentrating] in the ten weeks."<br>"[I found the yoga intervention] sometimes confrontational. You are focusing on yourself. I try to distract myself very often. I just turn on music and things like that. But now it was completely quiet and [it was all about] what is nice and not nice for you. […] That was good, I think, but also difficult to be focusing on your own feelings."
**Affective Domain**<br>Agitation or Irritability	12	20.69	"I found it [the yoga intervention] frustrating, because I used to be more flexible and things went easier. I found it frustrating that I was suffering and in pain. Not pain of 'I'm dying' but pain that it's just not nice. But yes, I am also stubborn and I do it [the painful posture] anyway."<br>"During the training I often felt frustrations about things not working out for me. I found that confrontational and less enjoyable."<br>"I noticed in myself that my body became very restless and my legs […]. I couldn't lie down anymore. I felt 'well, this is nice for a few times, but it doesn't suit me to continue with yoga'."<br>"Once, twice [going to a yoga class was] nice, but after that it is no longer relaxing for me. I'd rather go for a run or something like that."<br>"It took too long for me. It made me very restless. [...] I was busy [in my mind] with other things [...]. I found out that [it is better for me to] be active in order to relax than [do] yoga."<br>"I couldn't handle the peace in the training. I noticed that I became more restless from the quiet [atmosphere] and had trouble staying seated."<br>"Only half way [through the yoga class ...] it got very difficult for me. I couldn't stay quiet anymore. Because then you could start thinking a bit in your mind again and that's the point where things go wrong for me. I can no longer focus on resting. That was difficult for me."<br>"Some things [from the yoga intervention] don't suit me. One time [during yoga class] I got very frustrated about something and thought it didn't work […]."<br>"It [the yoga intervention] was mostly lying still [which I didn't like] and watching your breathing for ten minutes and things like that. In my mind, I had [thoughts] like 'aren't we finished yet?'."<br>"I didn't calm down in that group. I always came away very worked up with a lot of irritations and things. I couldn't concentrate on anything [during the yoga class]. I heard all kinds of things around me and I get annoyed by the people in the group. [I got annoyed with] little things. That you all sit there and one person gets up three times to fill her water bottle or something. I think 'everyone is here to rest now' and I can be very annoyed, but I don't say it."<br>"[I had a hard time] paying attention to myself and being with myself. I was always looking around and restless."

			“If we had to sit still or in a certain position [I felt …] 'we can do something else'. Then I started thinking too much. [I felt] restless.”<br>“Because you had to be very calm and very quiet [during the yoga class] and at a certain point you only heard yourself, that really pissed me off.”<br>“I'm quite a busy person myself, and I got very itchy from sitting.”<br>“Yoga was really quiet and calm and a bit airy-fairy. I had done it a few times and thought 'that's really not for me'. I don't like that extremely calm zen-ish [atmosphere]. That does not bring me relaxation [...]. Quite the contrary. Breathing. That's not for me.”<br>“[The yoga class] just made me impatient.”<br>“With yoga I [cannot] relax. If you have to meditate, then I can't.”<br>“There were moments during [the yoga sessions] where it was a bit quieter, but in general I noticed that it didn't create a lot of peace for me.”
**Somatic Domain**<br>Inflexibility	7	12.07	“Sometimes [I found the exercises] difficult because I am overweight. This made some movements less easy.”<br>“I didn't do much with yoga because I have many physical issues and [I] couldn't do many things.”<br>“Physically, [it was] very difficult […] at times. I couldn't participate in some things.”<br>“Physically, I sometimes had limitations.”<br>“[I found it] difficult [because] I'm not very flexible, so I couldn't do everything.”<br>“Some of the postures you had to do, I thought I could do it. But it turned out to be more difficult. I found that annoying. Because I'm someone who wants to do it immediately right. That didn't work and I found that difficult.”<br>“[…] I found some exercises physically difficult. For example, I couldn't touch my toes with my fingers.”<br>“I noticed that I wasn't that flexible. That some things didn't work out physically. That it's okay and you shouldn't get angry with yourself if it doesn't work out. In the beginning I could not do that [be ok with it]. But later it really got better.”
**Somatic Domain**<br>Pain	6	10.34	“Sometimes my hips or my back bother me. If I had to lie down my lower back would hurt very quickly. So, I couldn't keep my legs extended any longer, but I had to bend my knees, because otherwise my back hurt too much.”<br>“[I got] sore muscles. I was someone who never sported, so something like that makes me feel that I don’t want to go anymore.”<br>“[My] lower back is in a lot of pain when I lay down.”<br>“I had sore muscles.”<br>“I've always had neck problems and that got worse [because of the yoga training].”<br>“I had some muscle soreness at times.”
**Affective Domain**	6	10.34	“[We did an exercise in which] you bend over and have to let go and sigh very loud. I just couldn't do that. Because I felt ashamed. I struggled with that.”<br>“That loud breathing and noise making was striking to me. Everyone around me made the sound but I was more withheld.”

Self-Conscious Emotions			"It's uncomfortable that you're sitting there [in the yoga class] with all women who have depression. Because everyone was so quiet and calm and depressed. I always thought this was a very awkward moment."<br>"[The yoga class] was a social thing because everyone started doing it at the same time. But [there was] no real talking, so [it] actually felt really awkward."<br>"There was an exercise [...where you] had to [...] inhale and then say 'huh'. Almost nobody did it. I've done it sometimes. It was uncomfortable, but it was also a nice feeling to notice that if I did it, others did it too."<br>"[During the training] you were with other people, who had the same thing as you [depression]. I'm someone who doesn't really like to talk about how I feel and how it is. Sometimes [...in the yoga class] you are asked how you are doing and how things are. When I heard someone else talking, I thought 'I have that too', but I didn't dare to say that. [It felt] confrontational where others share what's the matter and you don't dare to share that and think 'that's just the way it is'."<br>"I haven't really talked to other participants. I don't really feel the need to. You come in [to the yoga class] and eight of these other depressed girls are sitting on the floor and it feels really bad to be part of that. [...] I'm a depressed young woman myself. When I came into the clinical setting [of the yoga class] I felt first: 'there's something wrong with me'. And second: 'that [yoga class] is with a group of people I don't want to belong to. Because I don't want to belong to a group that has problems or who are pathetic […]."<br>"It is always silence [at the start of the yoga session]. That in itself is fine because then you can sit in peace. But you feel very watched when you come in. Everyone is watching you and everyone is already seated."<br>"You sit there [during the yoga class] and are still for a very long time. I wasn't comfortable [with that]. For me there may [be] more fun. Then it would not have been hard for me [to be in the yoga class]. Because I had trouble with being quiet for an hour long."
**Somatic Domain** Fatigue or Weakness	2	3.45	"[I found it] tiring rather than relaxing."<br>"I was especially very tired after [the yoga session]. An hour and a half are quite long. It's stressful to be [with] a group of people you don't know every week. You are quite depressed. It takes a lot of effort to go there [to the yoga class], being with people. When you're depressed you don't really have the energy to do anything. You get really tired of [... the yoga intervention]."
**Somatic Domain** Breathing Changes	1	1.72	"I have breathing problems. That came forward very much [during the yoga intervention]. If I have to pay attention to my breathing, it becomes very difficult for me."
**Conative Domain**	1	1.72	"I remember that I unexpectedly became very competitive, while [yoga] is not a competitive thing. I got quite distracted because [when the yoga class] wasn't that physically [challenging], I went to look at the people I could see [around me]. 'She can stretch just as far as I can: let's see if I can go just a little further. Let's see if I can hold [the posture] just a little longer than everyone else.' So, I got strangely competitive."

Change in Effort or Striving			
**Affective Domain** Rage, Anger, or Aggression	1	1.72	“I've had anger and aggression in my body because of [a] trauma, which I couldn't handle very well. That yoga only made it more intense and [that …] made me very aggressive. I would come home [from the yoga class] and attack my parents or my brother. I was really, really worked up and aggressive.”
**Affective Domain** Re-experiencing of Traumatic Memories or Affect Without Recollection	1	1.72	“You are so relaxed [during the yoga session] that you have to let your mind wander and relax while doing that. If you let your thoughts run free, I envisioned everything that was traumatizing in my life. Your eyes had to be closed. I started to think even more. You could also see images clearly in front of you. I think any person with unprocessed trauma who goes into meditation, at least for me, they get a lot of images [coming up] in front of them. It was very confrontational.”
Other	1	1.72	“To close your eyes, I find that very unpleasant and unsafe. That is a thing for me. I feel like I'm losing my balance [when I close my eyes], especially when people are standing around me. I think 'I'm losing my balance' and [feel like I] can fall over or sink into the ground at any moment. I feel all kinds of movements that are not there and that feels very unsafe.”
